# Hierarchically Porous Wearable Composites for High‐Performance Stretchable Supercapacitors

**DOI:** 10.1002/advs.202500835

**Published:** 2025-04-25

**Authors:** Jing Han, Bingang Xu, Cuiqin Fang, Juyang Wei, Zihua Li, Xinlong Liu, Yujue Yang, Qian Wang, Junze Zhang

**Affiliations:** ^1^ Nanotechnology Center School of Fashion and Textiles The Hong Kong Polytechnic University Kowloon 999077 Hong Kong

**Keywords:** porous microstructure, stretchable, supercapacitor, wearable composite

## Abstract

With the rapid development of wearable electronic devices, the demand for flexible, durable, and high‐performance energy storage systems has increased significantly. Nevertheless, maintaining stable electrochemical performance during stretching while ensuring high stretchability and mechanical stability remains a challenge. Herein, this study proposes a novel type of stretchable supercapacitors made from carbon nanotube (CNT) and styrene‐butadiene‐styrene (SBS) composite scaffolds prepared on pre‐stretched carbon fabrics using the breath figure method. Hydrothermal treatment is then performed to grow NiCo‐LDH at the treated carbon fabrics. This method induces the formation of a hierarchically porous structure under high humidity conditions, controls the hydrothermal growth of NiCo‐LDH in the CNT/SBS composite scaffold, and significantly enhances the electrochemical performance and mechanical stability. The supercapacitor demonstrates remarkable retention of 94% capacitance under 80% tensile strain and sustains a small 8% degradation over 20 000 charge–discharge cycles, achieving a specific capacitance of 4948 mF cm⁻^2^ at 2 mA cm⁻^2^. The device has an energy density of 801.6 µWh cm⁻^2^ (400.6 Wh kg⁻¹) and exhibits excellent performance at a power density of 3.5 mW cm⁻^2^ (1749.5 W kg⁻¹). These properties make the supercapacitors a potential for next‐generation smart wearables and wearable electronics.

## Graphical abstract

This work presents hierarchically porous Nickel‐c oblate layered double hydroxide @ styrene‐butadiene‐styrene and carbon nanotubes composites for high‐performance stretchable supercapacitors, showing retention of 94% capacitance under 80% tensile strain and specific capacitance of 4948 mF cm⁻^2^ at 2 mA cm⁻^2^. The device has an energy density of 801.6 µWh cm⁻^2^ and a power density of 3.5 mW cm⁻^2^, offering great promise for next‐generation wearable electronics.

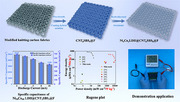

## 1 Introduction

The rapid adoption of wearable electronic devices, including health monitors, e‐textiles, and fitness trackers, has significantly increased the demand for flexible, stretchable, and durable energy storage systems.^[^ ^1^, ^2^ ^]^ Supercapacitors have garnered considerable attention among various energy storage technologies owing to their high‐power density, fast charge–discharge capability, and long cycle life.^[^ ^3^, ^4^ ^]^ However, integrating traditional supercapacitors with wearable devices is challenging because these devices tend to lose energy storage capacity when subject to mechanical deformations such as bending, twisting, or stretching.^[^ ^5^, ^6^, ^7^ ^]^ Therefore, stretchable supercapacitors that can maintain their electrochemical performance under deformation have become a focal point of research.

To meet the stringent mechanical and electrochemical requirements of wearable electronics, advanced material matchings, and innovative structures have been designed to provide flexibility and energy storage. Carbon‐based materials,^[^ ^8^ ^]^ particularly carbon nanotubes (CNTs)^[^ ^9^ ^]^ and carbon fibers,^[^ ^10^ ^]^ have become indispensable in this field owing to their superior electrochemical double‐layer (EDLC) energy storage nature, mechanical strength, and structural versatility. However, the energy storage capacity of carbon‐based materials still needs to be improved due to the ion adsorption and desorption characteristics of EDLC. When combined with pseudocapacitive materials like transition metal oxides (e.g., MnO₂) or conductive polymers (e.g., polyaniline), these hybrid systems can achieve higher energy storage capacity while maintaining the mechanical flexibility of carbon‐based substrates.^[^ ^11^, ^12^ ^]^ For instance, fabric‐shaped supercapacitors made from a rubber‐like stretchable hybrid CNT film exhibited remarkable stretchability, retaining their capacitance under strains as high as 134%.^[^ ^13^ ^]^ Flexible fiber‐shaped CNT supercapacitors have improved the performance of elastomeric supercapacitors with a coiled MnO_2_‐biscrolled CNT yarn.^[^ ^14^ ^]^ Despite these hybrid materials providing good flexibility, they still suffer from decreased capacitance under repeated or extreme stretching.^[^ ^15^ ^]^ Additionally, although pseudocapacitive materials effectively enhance energy storage, the properties tend to degrade over time due to cycling fatigue, especially in highly stretchable systems. Therefore, achieving high electrochemical performance while maintaining mechanical durability under high continuity and extreme strain remains unresolved.

Nickel‐cobalt layered double hydroxide (NiCo‐LDH) has emerged as a superior pseudocapacitive material to conventional transition metal oxides or conductive polymers.^[^ ^16^, ^17^, ^18^ ^]^ Owing to highly efficient faradaic activity, NiCo‐LDH offers some active sites for redox reactions to boost energy storage capacity. Additionally, the layered structure of NiCo‐LDH facilitates ion transport, improving charge storage efficiency. Compared with other transition metal oxides, NiCo‐LDH also exhibits better mechanical flexibility, making it an ideal pseudocapacitive material candidate for stretchable supercapacitors. For instance, a represented stretchable supercapacitor composed of MnO₂‐deposited nylon/CNT fibers exhibited poor cycling stability, reduced conductivity, and a 15% decrease in capacitance when stretched to 150% strain.^[^ ^19^ ^]^

In contrast, the combination of NiCo‐LDH with Ti_3_C_2_T*_x_* enhances the electrochemical performance and significantly improves mechanical stability, particularly under dynamic deformation conditions.^[^ ^20^, ^21^ ^]^ Recently, hierarchical porous structures have been proven to effectively increase the electrode surface area,^[^ ^22^, ^23^, ^24^ ^]^ thereby improving both charge storage capacity and ion diffusion, while maintaining excellent mechanical flexibility of the electrode system. Additionally, stretchable supercapacitors may be able to regain their electrochemical properties after being stretched. This would extend their useful life under high continuity and extreme strain.^[^ ^25^, ^26^ ^]^

Herein, styrene‐butadiene‐styrene (SBS) was combined with CNTs for casting onto pre‐stretched carbon fabric using the breath figure (BF) method under high‐humidity conditions. This BF technique utilizes evaporative cooling to induce the condensation of water droplets on the material's surface, which then self‐assemble into an ordered microstructure that acts as a dynamic template. The unique process resulted in a hierarchically, buckled, porous structure on the surface of the carbon fabric, which is crucial for the hydrothermal growth of NiCo‐LDH. A novel stretchable electrode with hierarchical porous architecture was formed by using CNT/SBS as the stretchable skeleton for NiCo‐LDH loading, achieving a synergistic improvement in the electrochemical performance and stretchability of the stretchable supercapacitor. Under an 80% tensile strain, the supercapacitor retains 94% of its initial capacitance, showcasing excellent mechanical resilience and energy storage stability. In cyclic stability tests, the device demonstrated less than 8% degradation in capacitance over 20 000 charge–discharge cycles, further highlighting its long‐term durability. Additionally, the specific capacitance of the device reaches up to 4948 mF cm⁻^2^ at 2 mA cm⁻^2^, illustrating superior energy storage capability compared to conventional flexible energy storage systems. The combination of high flexibility, stable electrochemical properties, and strong cycling stability under dynamic conditions makes this stretchable supercapacitor a competitive candidate for next‐generation wearable technology applications such as health monitoring and smart textiles.

## 2 Results and Discussion

**Figure 1:**
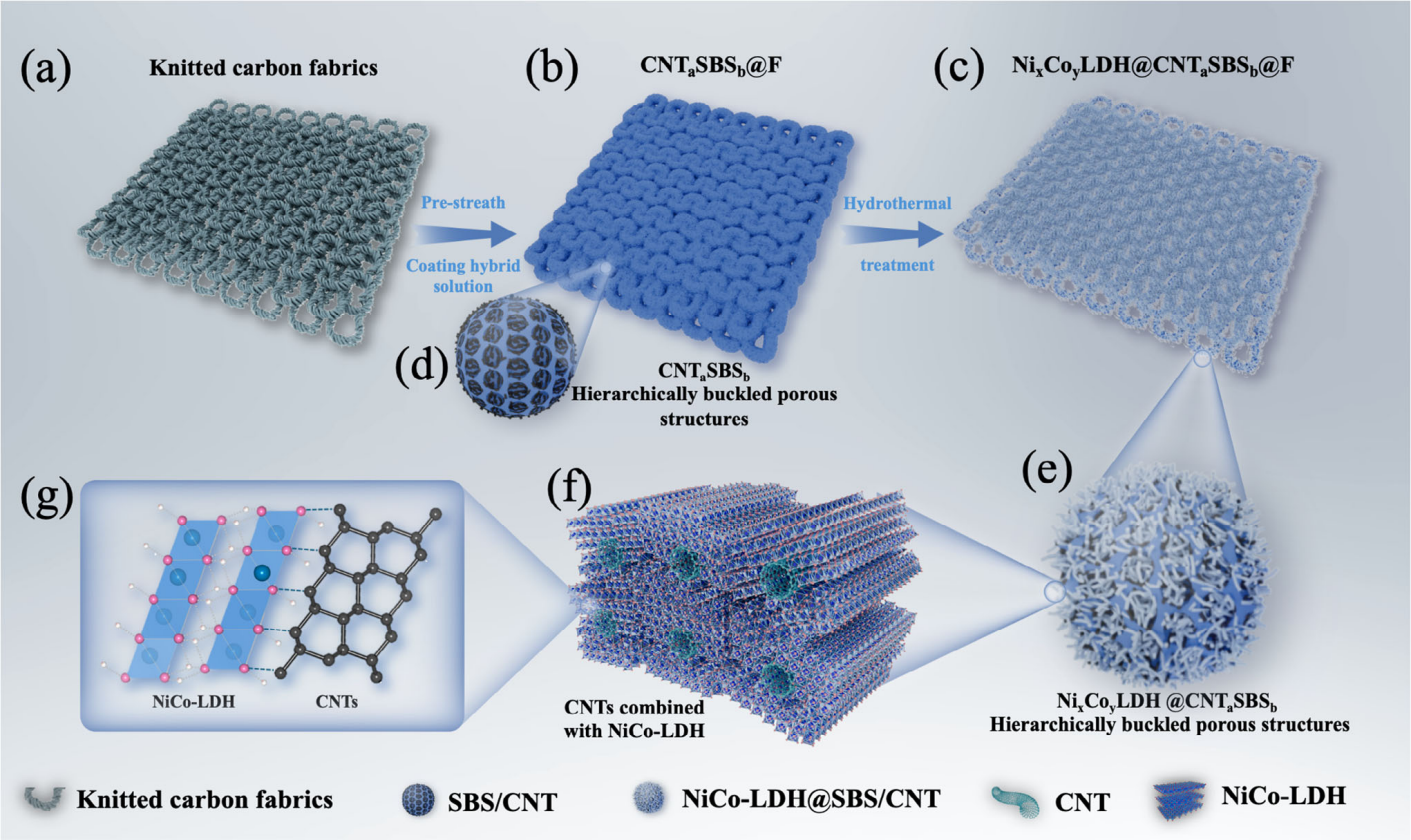
Schematic and mechanism illustration of the formation process of Ni_x_Co_y_ LDH@CNT_a_SBS_b_@F. a) Modified knitting fabrics. b) Hierarchically buckled porous structure formation of CNT_a_SBS_b_ on the knitted fabric. c) Ni_x_Co_y_ LDH growth on the CNT_a_SBS_b_@F. d) CNT_a_SBS_b_@F magnified image. e) Ni_x_Co_y_ LDH@CNT_a_SBS_b_@F magnified image. The schematic molecular model f) and chemical structure g) of CNTs combined with NiCo‐LDH.

In the preparation process of the supercapacitor, to enhance the stretchability and flexibility of the carbon fabrics, the carbon fiber was initially knitted into the carbon fabrics (**Figure** **1**a). This knitted fabric involved forming a flexible 2 × 2 twill structure through a knitted carbon fiber arrangement, which enhanced the material's ability to deform under external forces and allowed it to endure more significant levels of stretching and bending. To obtain a large specific surface area and hierarchically buckled porous structure fabrics (HBPFSs), the pre‐stretched carbon fabrics were coated with a mixture of different radio of SBS/CHCl_3_ and CNT/CHCl_3_ solutions (named CNT_a_SBS_b_@F) and processed under a high‐humidity environment using the BF Method^[^ ^27^, ^28^ ^]^ (Figure 1b). This technique utilizes evaporative cooling to induce the condensation of water droplets on the material's surface. These water droplets self‐assemble into an ordered microstructure, acting as a dynamic template that facilitates the formation of an ordered porous network of SBS/CNT on the fabric surface (Figure 1d). The elasticity of SBS provides the composite material with excellent durability and mechanical stability,^[^ ^29^ ^]^ while CNT establishes a continuous conductive pathway.^[^ ^30^ ^]^ Following the formation of the porous conductive fabric, hydrothermal treatment was applied to induce the growth of different content ratios of nickel‐cobalt layered double hydroxide (NiCo‐LDH) nanostructures (named Ni_x_Co_y_ LDH@CNT_a_SBS_b_@F) (Figure 1c). NiCo‐LDH is recognized for its considerable electrochemical activity and ion exchange properties. Through hydrothermal synthesis, NiCo‐LDH nanosheets are uniformly deposited across both the surface and inner pores of the SBS/CNT porous composite (Figure 1e), resulting in a hierarchical structure that further enhances the material's specific surface area and active sites.^[^ ^31^, ^32^ ^]^ In the molecular and chemical structure of CNTs combined with NiCo‐LDH (Figure 1f,g), the distinctive configuration of NiCo‐LDH nanowires effectively facilitates charge transfer and accelerates ion diffusion,^[^ ^33^, ^34^ ^]^ which improves the capacitor's fast charge–discharge performance. Additionally, the mechanical interlocking provided by the porous network substantially strengthens the adhesion between NiCo‐LDH nanosheets and the substrate, ensuring stability and minimizing detachment throughout repeated charge–discharge cycles.^[^ ^35^, ^36^ ^]^

SEM images are used to track the hierarchical porous structures of carbon fabrics with varying ratios of SBS/CHCl₃ and CNT/CHCl₃ solutions. At a 1:1 ratio of SBS/CHCl₃ to CNT/CHCl₃ solutions to form CNT_1_SBS_1_ @ F (Figure S1a,b, Supporting Information), the SBS matrix dominates the formation of the porous structure through the breath figure template, resulting in a relatively regular and uniform porous network with large and evenly distributed pores across the carbon fabric surface. The low CNT content at this ratio does not significantly impact the pore formation, as SBS remains the primary factor in stabilizing the structure. The SBS matrix provides sufficient support to maintain a smooth and consistent porous structure, critically for subsequent material deposition. When the ratio of SBS/CHCl₃ to CNT/CHCl₃ increases to 1:2, the pore size decreases, and the structure of CNT_1_SBS_1_ @ F becomes more densely packed (Figure S1c,d, Supporting Information). While the breath figure method is still effective at this ratio, the reduced SBS content leads to less stabilization of the pore network, making the structure slightly less orderly than the 1:1 ratio. The CNTs begin to play a more prominent role in forming the backbone of the porous structure, but the reduction of the SBS matrix compromises the regularity of the pores. When the ratio of SBS/CHCl₃ to CNT/CHCl₃ increases further to 1:3 (Figure S1e,f, Supporting Information), the SBS matrix on the carbon fabrics is no longer sufficient to stabilize the breath figure process. The excess CNT content destabilizes the solution, disrupting the water droplet template and resulting in irregular or absent pores. These results suggest that a proper balance between SBS/CHCl₃ and CNT/CHCl₃ solutions is crucial for achieving a stable and regular porous network. Excessive CNT content, while potentially increasing electrical conductivity, can negatively impact pore formation, which may affect ion diffusion and charge transport.

**Figure 2:**
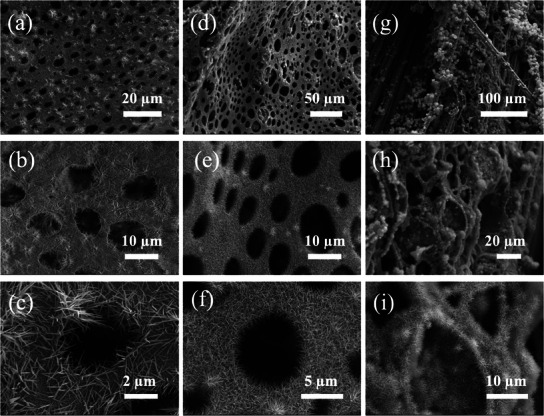
SEM images of NiCo‐LDH on the carbon fabric cast with different SBS and CNT ratio solutions at different magnifications. a–c) Ni_20_Co_40_ LDH @ CNT_1_SBS_1_ @ F, d–f) Ni_20_Co_40_ LDH@CNT_2_SBS_1_@F, and g–i) Ni_20_Co_40_ LDH@CNT_2_SBS_1_@F.

The NiCo‐LDH growth on carbon fabrics with different ratios of SBS/CHCl₃ and CNT/CHCl₃ solutions after hydrothermal treatment is also studied with SEM images (**Figure** **2**). Figure 2a–c represent the SEM images of Ni_20_Co_40_ LDH@CNT_1_SBS_1_@F, where large and evenly distributed pores are observed on the carbon fabric surface, but NiCo‐LDH growth is relatively sparse, indicating that the available CNT nucleation sites are limited at this ratio. The lower CNT content does not provide sufficient active sites for extensive NiCo‐LDH deposition. The Ni_20_Co_40_‐LDH @ CNT_2_SBS_1_@ F shows denser and more uniformly distributed NiCo‐LDH growth. In Figure 2d, the increased CNT content provides more nucleation sites for NiCo‐LDH growth, resulting in a denser network of nanowires across the porous structure. The enhanced distribution of NiCo‐LDH nanowires in Figure 2e further demonstrates that a higher CNT content can improve the material's surface area, making it more favorable for electrochemical reactions. Figure 2f illustrates that the NiCo‐LDH layer covers a significant portion of the carbon fabric with a well‐developed nanowire structure, suggesting more electrochemically active sites than the lower CNT content samples. However, at the highest CNT/CHCl₃ to SBS/CHCl₃ ratio of 3:1, the Ni_20_Co_40_ LDH @ CNT_3_SBS_1_ @ F exhibits an irregular porous structure. As seen in Figure 2g, the excess CNT content disrupts the breath figure template, leading to the collapse of the pore structure and uneven growth of NiCo‐LDH. Figure 2h,i show that NiCo‐LDH aggregates on the surface, forming clumps that may hinder uniform ion transport. The destabilized pore structure and irregular nanowire growth can potentially reduce the material's electrochemical performance, as the distribution of active sites becomes less optimal. In conclusion, the Ni_20_Co_40_ LDH@CNT_2_SBS_1_@F exhibits the best porous structure and the most uniform NiCo‐LDH growth. The higher CNT content provides more growth sites, resulting in a denser nanosheet structure, which is expected to enhance electrochemical performance.

The influence of Ni and Co concentrations on the morphology of NiCo‐LDH growth on the CNT_2_SBS_1_ @ F substrate is further investigated. At equal concentrations of Ni^2^⁺ and Co^2^⁺ (20 mm each, Figure S2a,b, Supporting Information), the Ni_20_Co_20_ LDH @ CNT_2_SBS_1_ @ F shows well‐ordered NiCo‐LDH nanowires forming across the porous structure. The uniform distribution of these nanowires creates heavy active sites for electrochemical reactions, and the porous network remains intact, which is crucial for effective ion transport.^[^ ^32^ ^]^ As the Co concentration increases to 40 mm (Figure S2c,d, Supporting Information), the sample Ni_20_Co_40_ LDH @ CNT_2_SBS_1_ @ F exhibits denser nanowire growth, with smaller pore sizes, enhancing the surface area available for electrochemical activity. The compact arrangement of these nanowires provides an ideal balance between maintaining the porous structure and maximizing the number of active sites. In contrast, when the Ni^2^⁺ concentration is increased to 40 mm (Figure S2e,f, Supporting Information), the excessive NiCo‐LDH Ni_40_Co_20_ LDH on the CNT_2_SBS_1_ @ F shows a nanosheet architecture instead of a nanowire structure, almost completely covering the original porous substrate. This dense layer of nanosheets obscures the underlying pore network, potentially reducing the accessibility of the electrochemically active sites and hindering ion diffusion. These results highlight the importance of controlling the Ni^2^⁺ and Co^2^⁺ concentrations to preserve the porous architecture while optimizing the nanostructure morphology for electrochemical applications. In addition, SEM images (Figure S3, Supporting Information) of Ni_20_Co_40_ LDH@CNT_2_SBS_1_@F show that NiCo‐LDH forms uniform, densely packed nanowires on CNT/SBS, while it grows irregularly and aggregates on pure knitted carbon fabric. CNT provides nucleation sites, and SBS enhances adhesion, ensuring even deposition. This structure stabilizes NiCo‐LDH, preventing nanowire detachment during mechanical deformation. EDX elemental mappings confirm the uniform distribution of Ni, Co, and O throughout the CNT_2_SBS_1_@ F substrate. Figure S4a–c (Supporting Information) correspond to the samples Ni_20_Co_20_ LDH @ CNT_2_SBS_1_ @ F, Ni_20_Co_40_ LDH @ CNT_2_SBS_1_@F, and Ni_40_Co_20_ LDH@CNT_2_SBS_1_@F, respectively. The elemental mappings demonstrate that Ni and Co are evenly distributed across the surface of the porous structure, ensuring that the NiCo‐LDH nanowires/nanosheets are uniformly integrated throughout the substrate.

**Figure 3:**
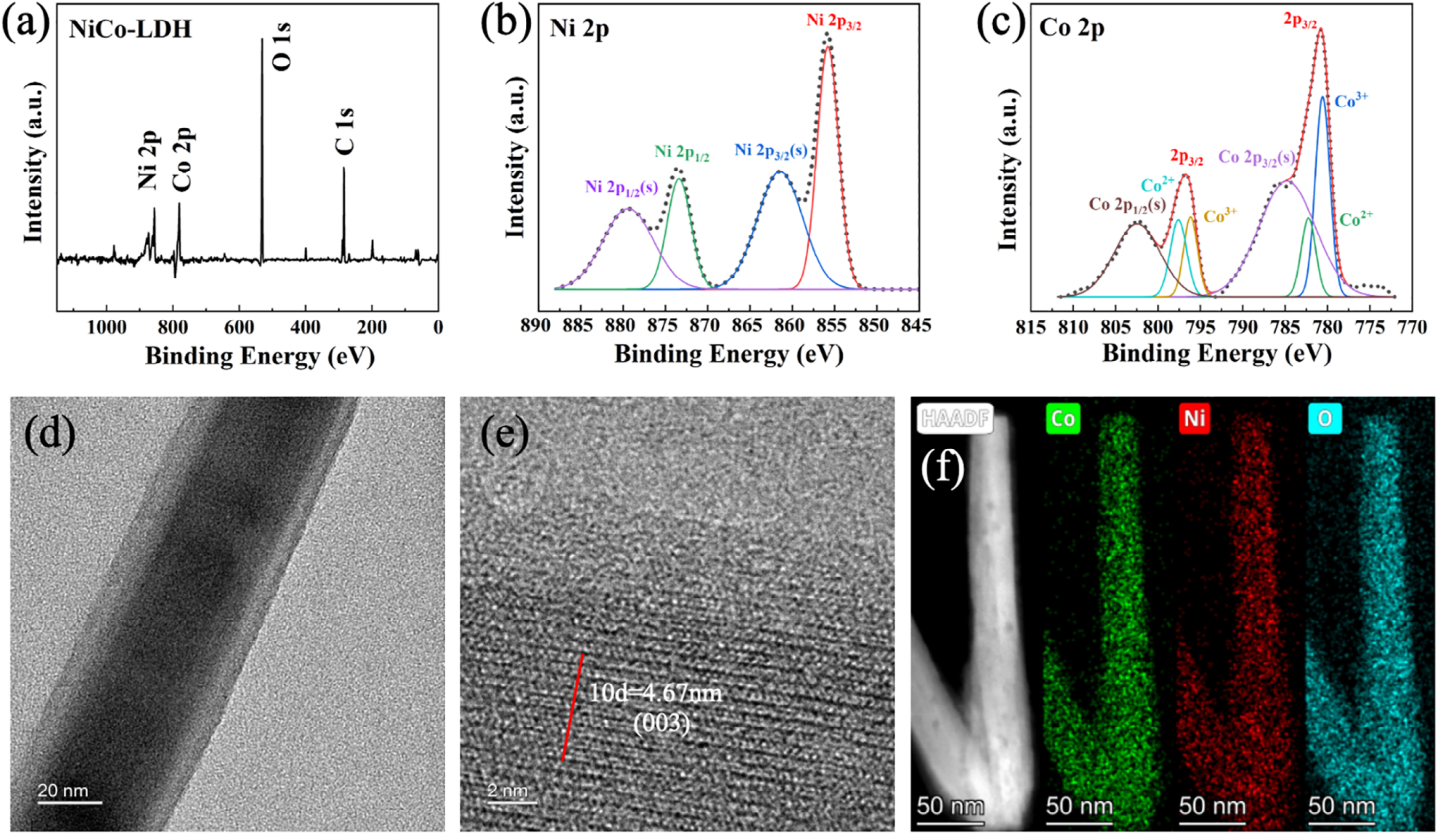
a) XPS survey spectrum of the NiCo‐LDH sample. b) High‐resolution XPS spectrum of Ni 2p. c) High‐resolution XPS spectrum of Co 2p. d) TEM image of NiCo‐LDH. e) HR‐TEM image of the NiCo‐LDH structure. f) Elemental mapping displays the Ni, Co, and O distribution in the nanostructure.

X‐ray photoelectron spectroscopy (XPS) is used to analyze the elemental composition and chemical states of the as‐prepared NiCo‐LDH of Ni_20_Co_40_ LDH@CNT_2_SBS_1_@F. In the overall survey spectrum (**Figure** **3**a), distinct peaks corresponding to Ni 2p, Co 2p, O 1s, and C 1s confirm the presence of Ni and Co, verifying the successful formation of NiCo‐LDH. High‐resolution XPS spectra further reveal detailed information about the oxidation states of Ni and Co. The Ni 2p spectrum (Figure 3b) displays two main peaks at binding energies of 873.5 and 855.3 eV, corresponding to Ni 2p_1/2_ and Ni 2p_3/2_, respectively. The satellite peaks and deconvoluted components also indicate the coexistence of Ni^2^⁺ and Ni^3^⁺ oxidation states. The Co 2p spectrum (Figure 3c) shows characteristic peaks at 796.4 eV (Co 2p_1/2_) and 781.4 eV (Co 2p_3/2_), with deconvolution revealing the presence of Co^2^⁺ and Co^3^⁺. The NiCo‐LDH structure is composed of a mixed valence of Ni and Co, which is crucial for enhancing the redox reactions in energy storage applications. The high‐resolution transmission electron microscopy (HR‐TEM) images provide further insights into the nano‐structural features of the NiCo‐LDH. Figure 3d shows a well‐defined nanowire structure, and the diameter is ≈40 nm. The lattice fringes observed in Figure 3e correspond to an interplanar spacing of 0.467 nm, indexed to the (003) plane of LDH. Figure S5 (Supporting Information) presents the X‐ray diffraction (XRD) pattern of Ni_20_Co_40_ LDH. The characteristic peaks at 11.3°, 33.5°, 34,4°, and 61.3° are detected, which are attributed to the crystal planes of (003), (101), (012), and (113), respectively.

Elemental mapping (Figure 3f) further confirms the homogeneous distribution of Ni, Co, and O throughout the nanowire. In addition, the Brunauer–Emmett–Teller (BET) results show that pure knitted carbon fabric has a negligible surface area (0.0237 m^3^ g^−1^) and no detectable Barrett–Joyner–Halenda (BJH) pore size distribution (Figure S6a, Supporting Information), confirming its non‐porous nature. SBS@F and CNT_2_/SBS_1_@F exhibit similar BET surface areas (0.845 m^3^ g^−1^) but different pore size distributions, indicating that an appropriate ratio of CNT to SBS significantly regulates the porous structure. After NiCo‐LDH growing, Ni_20_Co_40_@CNT_2_SBS_1_@F shows a substantial increase in BET surface area (10.315 m^3^ g^−1^) but almost no change in the pore size distribution, suggesting that NiCo‐LDH contributes additional accessible surface sites without significantly changing the fundamental pore structure (Figure S6b, Supporting Information).

**Figure 4:**
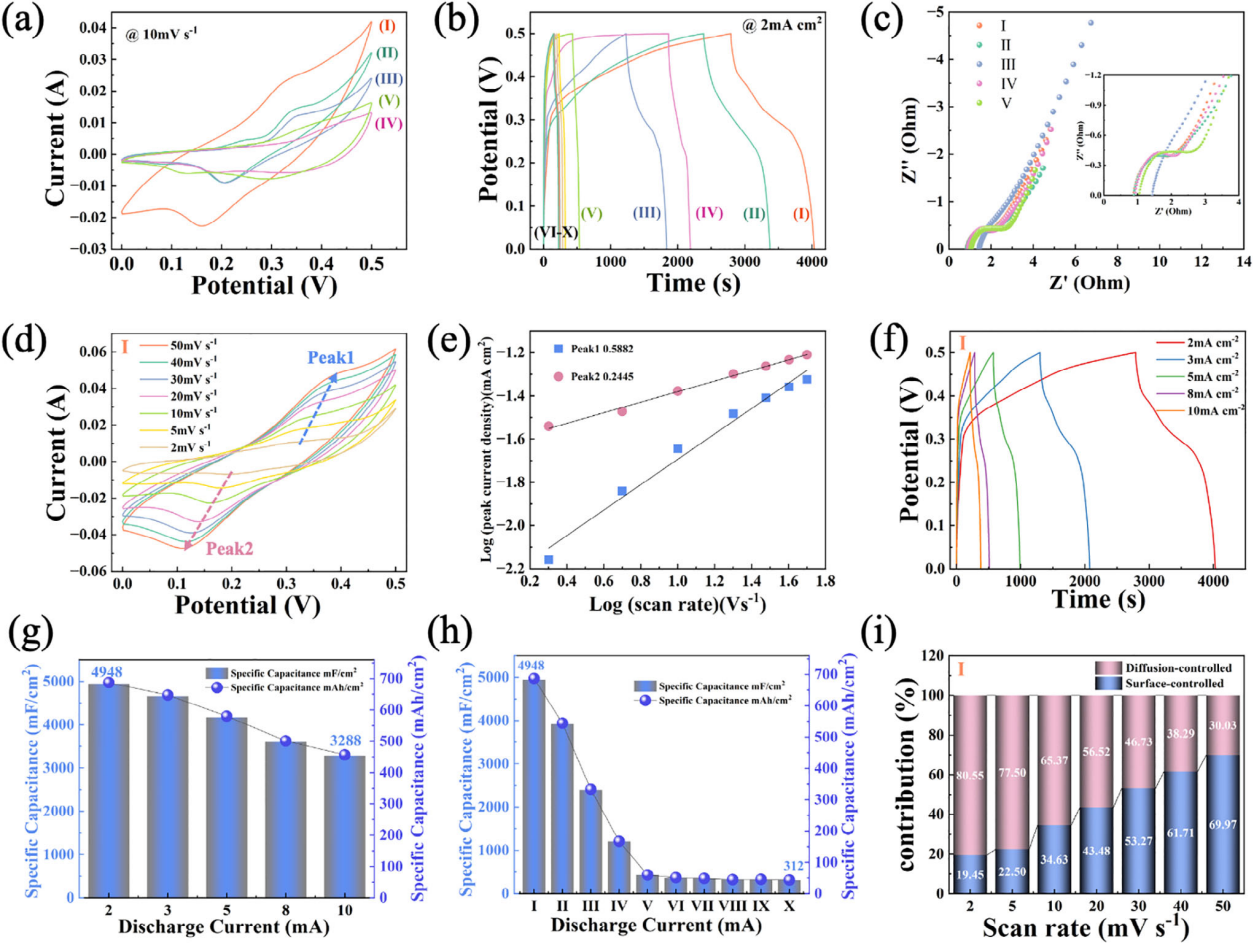
Electrochemical performance of Samples I‐X. a) CV curves of samples *I*–*V* at 10 mV s⁻¹. (b) GCD curves of samples *I*–*X* at 2 mA cm⁻^2^. c) Nyquist plots of samples *I*–*V*, the inset shows the magnified high‐frequency region. d) CV curves of Sample I at varying scan rates. e) Log‐log plot of peak current density versus scan rate for sample I. f) GCD curves of sample I at different current densities. g) Specific capacitance of sample I as a function of discharge current density. h) Specific capacitance of samples *I*–*X* at 2 mA cm⁻^2^. i) Contribution of diffusion‐controlled and surface‐controlled capacitance for Sample I at different scan rates.

The electrochemical performance of the prepared fabrics is systematically investigated by a standard three‐electrode system in a 3 m KOH solution, including cyclic voltammetry (CV), galvanostatic charge–discharge (GCD), and electrochemical impedance spectroscopy (EIS). In the three‐electrode system, a **saturated calomel electrode (SCE)** was used as the reference electrode, and a **graphite electrode** was employed as the counter electrode, with NiCo‐LDH@ CNTaSBSb @F as the working electrode. The NiCo‐LDH growth fabrics, including Ni_20_Co_40_ LDH@CNT_2_SBS_1_@F, Ni_20_Co_40_ LDH@CNT_1_SBS_1_@F, Ni_20_Co_40_ LDH@CNT_3_SBS_1_@F, Ni_40_Co_20_ LDH@CNT_2_SBS_1_@F, and Ni_20_Co_20_ LDH@CNT_2_SBS_1_@F are named as I, II, III, IV, and V, respectively. The fabrics without NiCo‐LDH growth, including pure knitted carbon fabric, CNT_3_SBS_1_@F, CNT_1_SBS_1_@F, SBS@F, and (X) CNT_2_SBS_1_@F are named as VI, VII, VIII, IX, and X, respectively. The CV curves (**Figure** **4**a; Figure S7, Supporting Information) for the as‐prepared fabrics at 10 mV s⁻¹ present rectangular shapes with obvious redox peaks, revealing the combination of EDLC characteristic of CNTs and pseudocapacitive nature of NiCo‐LDH. Among the NiCo‐LDH fabrics, the sample I exhibits the highest current response and the largest enclosed CV area, indicating the best electrochemical performance owing to its well‐defined, uniformly buckled, and porous structure, which facilitates the optimal growth of NiCo‐LDH nanowires. As the sample progresses to V, there is a noticeable reduction in both the current response and the CV area, signifying a gradual decrease in capacitance. This is due to the excessive dosage of NiCo‐LDH nanosheets with uneven distribution or insufficient NiCo‐LDH nanowires on the porous structure of the knitted fabric substrate. For the fabrics without NiCo‐LDH growth, the current response and the CV area are greatly reduced, becoming almost identical. These samples exhibit moderate current response and CV area owing to the inherent capacitive nature of pure knitted carbon fabric and CNTs. These results demonstrate that the electrochemical performance of the knitted fabrics is attributed to the appropriate CNTs and NiCo‐LDH distribution.

The GCD curves at 2 mA cm⁻^2^ of the fabrics are presented in Figure 4b and Figure S8 (Supporting Information). Sample I demonstrates the longest discharge time, reflecting its highest energy storage capacity. Meanwhile, its nearly symmetrical charge and discharge profiles suggest excellent Coulombic efficiency owing to the highly interconnected porous structure and reversible redox reactions of the NiCo‐LDH/CNT composite. As the sample progresses to V, there is a gradual reduction in the discharge time. For the samples without NiCo‐LDH growth, shorter discharge times are observed. In Figure 4c and Figure S9 (Supporting Information), all fabrics exhibit minor interceptions on the *x*‐axis and small semicircles in the high‐frequency region, indicating low internal resistance Rs and charge transfer resistance (Rct), along with high conductivity and efficient charge transport. Figure 4d and Figure S10 (Supporting Information) show the CV curves of all samples at various scan rates. As the scan rate increases, the redox peaks symmetrically shift toward more positive and more negative potentials, respectively, suggesting good electrochemical reversibility. At 2 mV s^−1^, two distinct redox peaks (Peak 1 and Peak 2) are observed, highlighting the pseudocapacitive behavior. Figure 4e further analyzes the fitting relationship between the two peak currents and scan rates in a logarithmic plot of sample I. Peak 1 exhibits a slope of 0.5882 and Peak 2 shows a slope of 0.2445, indicating that diffusion‐controlled process governs electrochemical behavior.

Figure 4f and Figure S11 (Supporting Information) present GCD curves of all samples at various current densities. As the discharge current increases, the discharge time decreases. Among the fabrics with NiCo‐LDH growth, sample I exhibits the longest discharge time at the same discharge current, indicating its highest charge storage capability. Compared with the fabrics with NiCo‐LDH, the fabrics without NiCo‐LDH growth exhibit shorter discharge times. Based on the GCD files, Figure 4g illustrates the specific capacitance of sample I at different discharge currents. At 2 mA cm⁻^2^, sample I demonstrates a maximum specific capacitance of 4948 mF cm⁻^2^, indicating its excellent charge storage capacity. As the discharge current increases to 10 mA cm⁻^2^, the specific capacitance gradually decreases to 3288 mF cm⁻^2^, reaching a high capacitance rate of 66.45%. Figure 4h compares the specific capacitance of all samples at 2 mA cm⁻^2^. Sample I exhibits the highest specific capacitance (4948 mF cm⁻^2^) among the fabrics with NiCo‐LDH growth, which is attributed to the optimized porous structure of the knitted fabrics and appropriate distributions of CNTs and NiCo‐LDH nanowires. For the samples without NiCo‐LDH, the specific capacitance values drop sharply. Figure 4i further illustrates the diffusion‐controlled and surface‐controlled contributions at various scan rates of sample I. At lower scan rates of 2 mV s^−1^, diffusion‐controlled contributions are ≈80.55%, indicating that ions have sufficient time to penetrate deep into the porous structure of the fabric and participate in the redox reactions. As the scan rate increases, surface‐controlled contribution becomes more prominent, reaching 69.97% at 50 mV s^−1^, reflecting that the charge storage is primarily confined to the electrode surface. These results demonstrate that sample I exhibits both diffusion‐controlled and surface‐controlled charge storage behaviors depending on the scan rate.

To evaluate the electrochemical performance of Ni_20_Co_40_@CNT_2_SBS_1_@F under tensile conditions, the electrochemical performance of the Ni_20_Co_40_@CNT_2_SBS_1_@F at different tensile strains of 0%, 50%, and 80% are tested in the three‐electrode system. From Figure S12a,b (Supporting Information), small variations of current response and discharge time are detected from the CV and GCD curves upon tensile strains of 0%, 50%, and 80%. As the tensile strain increases, the CV curves exhibit a minor shift, and the GCD discharge time slightly decreases. The Nyquist plots show that the impedance response slightly evolves under different tensile strain conditions (Figure S12c, Supporting Information). These are due to the variations in ion transport pathways and electrode‐electrolyte interface resistance under mechanical deformation. Despite these variations, the Ni_20_Co_40_@CNT_2_SBS_1_@F maintains a stable energy storage behavior across the entire strain range, with all curves retaining their characteristic shapes and well‐defined redox peaks. These results collectively indicate that though minor variations occur due to mechanical stretching, the overall electrochemical performance of Ni_20_Co_40_@CNT_2_SBS_1_@F remains robust, ensuring its reliable operation in stretchable energy storage applications.

**Figure 5:**
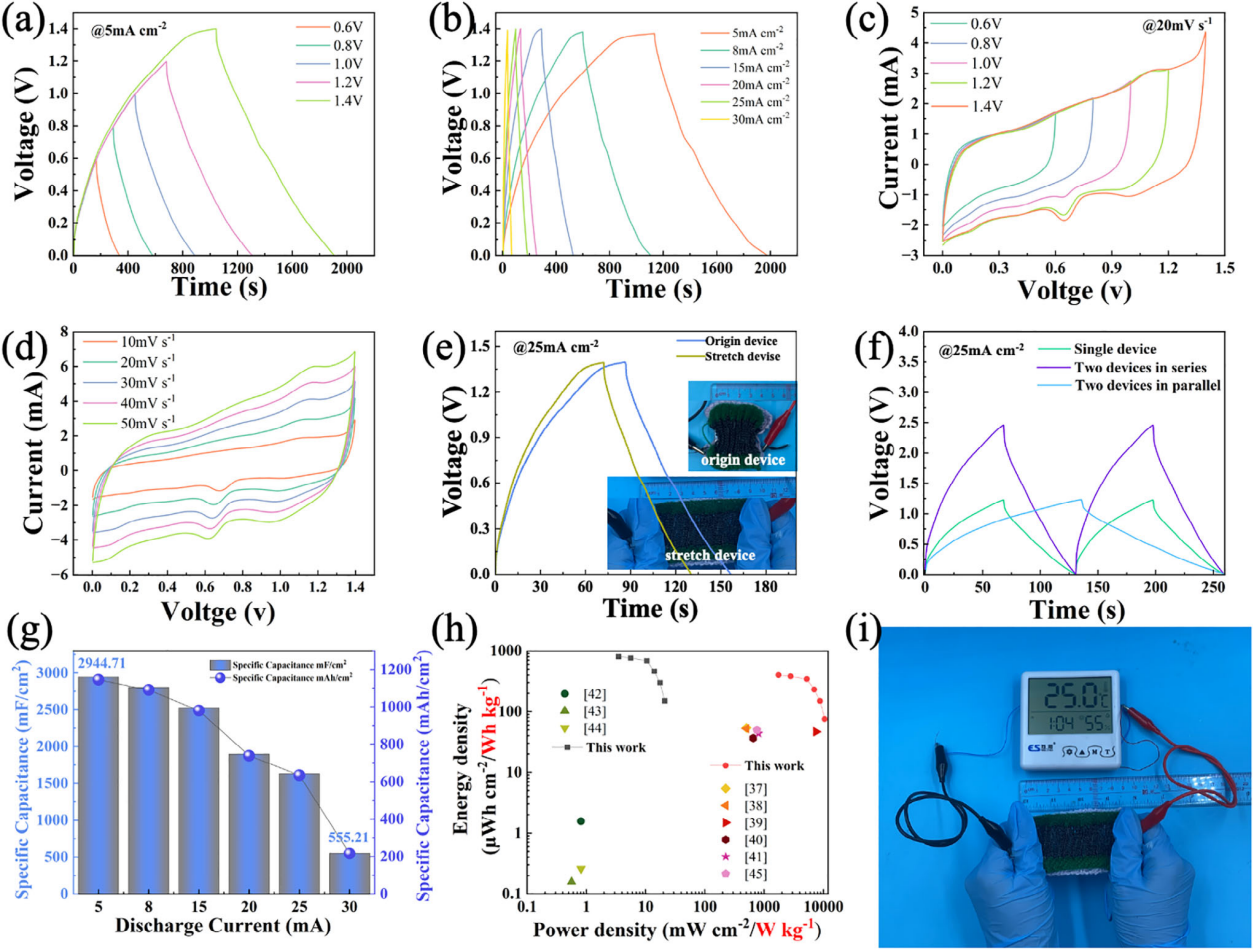
Electrochemical performance of the symmetric supercapacitor based on Ni_20_Co_40_ LDH@CNT_2_SBS_1_@F electrodes. a) Galvanostatic charge–discharge (GCD) curves at different voltages with a constant current density of 5 mA cm⁻^2^. b) GCD curves at varying current densities at 1 .4V. c) CV curves at different voltages and a scan rate of 20 mV s⁻¹. d) CV curves at different scan rates at 1 .4V. e) GCD curves at 2 mA cm⁻^2^, comparing the performance of the original device and the device under stretch (80%). f) GCD curves for a single device, two devices in series, and two devices in parallel. g) Specific capacitance as a function of discharge current density. h) Ragone plot showing the comparison of energy density and power density for the stretchable supercapacitor with the previously reported supercapacitors. i) Demonstration application.

A symmetric stretchable supercapacitor is further assembled to assess the practical applicability of Ni_20_Co_40_ LDH@CNT2_S_BS_1_@F. Figure S13 (Supporting Information) shows the assembly of the stretchable supercapacitor, where two Ni_20_Co_40_ LDH@CNT_2_SBS_1_@Fs are sandwiched with a gel electrolyte. Figure S14 (Supporting Information) shows that the potential windows of the Ni_20_Co_40_ LDH@CNT_2_SBS_1_@F as the negative electrode (0–0.5 V) and the positive electrode (−0.9–0 V). **Figure** **5**a shows the GCD curves of the symmetric supercapacitor at different voltage windows. The nearly symmetrical and linear charge–discharge curves demonstrate excellent reversibly capacitive behavior. As the voltage increases, the discharge time increases without obvious deformations, indicating the ability to store energy at higher voltage windows. Figure 5b illustrates the GCD profiles at various current densities. Figure 5c shows the CV curves at different voltage windows with a scan rate of 20 mV s⁻¹. The quasi‐rectangular shapes of the curves indicate typical capacitive behavior, along with redox peaks corresponding to the pseudocapacitive behavior, which are associated with the NiCo‐LDH/CNT composite. Figure 5d shows that the redox peaks on the CV curves slight shift at different scan rates, suggesting a stable energy storage behavior. Figure 5e compares the GCD curves at 2 mA cm⁻^2^ and CV and GCD curves at 10 mA cm^−2^ of the stretchable supercapacitors before and after stretching strain of 80%. The near‐identical curves demonstrate that the charge–discharge curve of the stretchable supercapacitor remains almost unchanged before and after mechanical deformation, demonstrating excellent flexibility and structural integrity.

To further demonstrate the electrochemical performance of the stretchable supercapacitor under tensile deformation, CV and GCD tests of the stretchable supercapacitor at different strain levels of 0%, 50%, and 80% are shown in Figure S15 (Supporting Information). The CV and GCD curves nearly maintain identical shapes upon different tensile strains, with consistent discharge durations, confirming the device's excellent electrochemical stability under tensile deformation. The specific capacitance (58.71 to 57.85mF cm^−2^)retention exceeds 98%, indicating that tensile strain does not significantly impact the specific capacitance. These results further validate that the stretchable supercapacitor composed of Ni_20_Co_40_@CNT_2_SBS_1_@F retains stable electrochemical performance under mechanical deformation, thus reinforcing its reliability in wearable applications. Figure S16 (Supporting Information) shows the SEM images of Ni_20_Co_40_@CNT_2_SBS_1_@F electrodes after stretching to different strain levels 0%, 50%, and 80% strain. The morphology remains highly stable, with NiCo‐LDH nanowires maintaining their structure and attachment to the CNT/SBS scaffold. Figure S17 (Supporting Information) shows the GCD curves at different bending angles (0°, 30°, 60°, 90°, and 180°) exhibit nearly identical charge–discharge profiles, with consistent discharge times and symmetrical shapes. This is attributed to the hierarchically porous structure of the knitted fabric, which enables the stretchable supercapacitor to maintain its high energy storage performance in the stretched state.

Figure 5f presents the GCD curves of a single device, two devices in series, and two devices in parallel at 25 mA cm⁻^2^. The series configuration increases the voltage, while the parallel configuration increases the current, demonstrating the device's modular capability to meet various energy and power requirements. Such flexibility in configuration is essential for scalable energy storage systems. Figure 5g shows the specific capacitance as a function of discharge current density. As the current density increases from 5 to 30 mA cm⁻^2^, the specific capacitance decreases from ≈2944.71 to 555.21 mF cm⁻^2^. The supercapacitor still demonstrates good rate capability, retaining a significant proportion of its capacitance even at high current densities. The relationship between the power density and the energy density for the NiCo‐LDH device and other reported counterparts are shown in Figure 5h. As the power density increases, the energy density decreases, a typical trade‐off for supercapacitors. The device achieves competitive energy density even at higher power densities, making it suitable for applications requiring high power and reasonable energy storage. The NiCo‐LDH device delivered a high energy density of 801.6 µWh cm^−2^ (400.6 Wh kg^−1^) at a power density of 3.5 mW cm^−1^ (1749.5 W kg^−1^), which was higher than its counterparts, such as LDH materials of 3D NiCo‐LDH microflowers derived from ZIF‐67 (53.31 Wh kg⁻¹ at 749.7 W kg⁻¹),^[^ ^37^ ^]^ Al‐doped NiCoP derived from NiCo‐LDH precursors (51.7 Wh kg⁻¹ at 500 W kg^−1^),^[^ ^38^ ^]^ GQDs pinned on NiCo‐LDH hollow micro‐tunnels (46 Wh kg⁻¹ at 7440 W kg^−1^),^[^ ^39^ ^]^ NiCo‐LDH microspheres derived from Ni‐MOF (36.1 Wh kg⁻¹ at 649 W kg⁻¹),^[^ ^40^ ^]^ NiCo‐LDH nanosheet array/Ag nanowire (42.9 Wh kg⁻¹ at 800 W kg⁻¹),^[^ ^41^ ^]^ NiCo‐LDH nanowires@nanosheets core‐shell (298.6 µWh cm⁻^2^ at 0.80 mW cm⁻^2^),^[^ ^42^ ^]^ yarn coated with Ni‐Co‐S (48.7 µWh cm⁻^2^ at 0.553 mW cm⁻^2^),^[^ ^43^ ^]^ Ni(OH)_2_ nanosheet wrapped NiCo_2_O_4_ on CNTF (103.8 µWh cm⁻^2^ at 0.8 mW cm⁻^2^),^[^ ^44^ ^]^ 3D hierarchical NiCo_2_O_4_@NiCo‐LDH (49 Wh kg^−1^ at 750 W kg^−1^).^[^ ^45^ ^]^ Moreover, Tables S1 and S2 (Supporting Information) shows a comprehensive comparison of our stretchable supercapacitor with the previously reported supercapacitors^[^ ^46^, ^47^, ^48^, ^49^, ^50^, ^51^, ^52^, ^53^, ^54^, ^55^ ^]^, highlighting the competitive electrochemical performance and superior mechanical flexibility of our device.

In contrast to typical capacitance retention values, the symmetric capacitor exhibits remarkable long‐term stability in both liquid and gel electrolytes. As shown in Figure S18 (Supporting Information), the capacitor in 3 m KOH solution achieves a coulombic efficiency of 95.06% after 5000 cycles and maintains 93.97% efficiency even after 20 000 cycles at 20 mA cm⁻^2^. Meanwhile, the symmetric capacitor of Ni_20_Co_40_ LDH@CNT_2_SBS_1_@F using a gel electrolyte retains 86.37% of its initial capacity after 5000 cycles, demonstrating enhanced durability and suitability for encapsulated solid‐state devices. To assess the supercapacitor's durability under mechanical deformation, we conducted a 5000‐cycle electrochemical stability test on the original device, devices subjected to 1000 bending cycles (180°) and 1000 stretching cycles (80% strain). In Figure S19 (Supporting Information), the coulombic efficiency of the original device remained stable throughout the cycling process, maintaining a high retention of 99.1% at the end of 5000 cycles. Similarly, the device measured to 1000 bending cycles before testing exhibited a slight decrease in coulombic efficiency to 97.5%, indicating excellent electrochemical stability despite prior mechanical deformation. Likewise, the device that underwent 1000 stretching cycles before testing retained 97.8% of its coulombic efficiency, further confirming the supercapacitor's robustness under tensile stress. Figure 5i shows a practical application demonstration of the stretchable supercapacitor, which powers an electronic thermo‐hygrometer under stretching. This demonstrates the real‐world applicability of stretchable supercapacitors in wearable electronic devices.

## 3 Conclusion

This study develops a stretchable supercapacitor with high flexibility and excellent energy storage performance to meet the energy needs of wearable electronic devices. The CNT/SBS composite scaffold, constructed under high humidity conditions using the BF method, forms an electrode with a hierarchical porous structure, which improves conductivity and surface area while providing support for the uniform growth of NiCo‐LDH. The experimental results show that the Ni_20_Co_40_ LDH@CNT_2_SBS_1_@F electrode reaches a capacitance of 4948 mF cm⁻^2^ at 2 mA cm⁻^2^, showing excellent energy storage capability. In addition, under 80% tensile strain, the device can maintain 94% of the initial capacitance; after 20 000 charge and discharge cycles, its capacitance decay is only 8%, proving its stability in repeated mechanical deformation and long‐term use. The energy density of the supercapacitor reaches 801.6 µWh cm⁻^2^ (400.6 Wh kg⁻¹), and it still shows excellent energy output efficiency at a power density of 3.5 mW cm⁻^2^ (1749.5 W kg⁻¹). These characteristics indicate that the composite material not only has broad application prospects in flexible and wearable devices but also provides a new solution for the design of high‐performance, stretchable energy storage devices in the future. This design idea lays the foundation for the development of energy storage devices with both high electric performance and high mechanical flexibility and is expected to promote the development of next‐generation wearable electronic devices and flexible energy systems.

## 4 Experimental Section

### Materials

The following materials were used for the experiments: polystyrene‐block‐polybutadiene‐block‐polystyrene (SBS), multi‐walled carbon nanotubes (MWCNTs), chloroform (CHCl₃), cobalt(II) sulfate hexahydrate (CoSO₄ 6H₂O), nickel(II) sulfate hexahydrate (NiSO₄ 6H₂O), potassium hydroxide (KOH), sodium dodecylbenzene sulfonate (SDBS), acrylamide, N,N′‐methylene‐bisacrylamide (BMAA), potassium persulfate (KPS), tetramethylethylenediamine (TEMED), and 3K carbon fibers were purchased from Sigma–Aldrich. All reagents were of analytical grade and used without further purification.

### Fabrication of Conductive Buckled Porous Carbon Fabrics

A 2 × 2 twill of 3K carbon knitted fibers was prepared to ensure sufficient elasticity of the fabric. The knitted carbon fabrics were then subject to solution casting. The casting solution was a mixture of SBS/CHCl₃ (200 mg mL^−1^) and CNT/CHCl₃ (40 mg mL^−1^) in varying ratios. The three ratios used for solution casting were 200 mg mL^−1^ SBS/CHCl₃: 40 mg mL^−1^ CNT/CHCl₃ = 1:1, 1:2, and 1:3. Before casting, the carbon fabrics were pre‐stretched to induce a buckled structure. The solutions were then cast onto the stretched carbon fabrics, allowing the formation of a buckled porous structure through the breath figure method under high‐humidity conditions (humidity >90%). After casting, the samples were dried completely at room temperature. The resulting samples were labeled based on the casting solution used: CNT_1_SBS_1_@F, CNT_2_SBS_1_@F, and CNT_3_SBS_1_@F.

### Preparation of NiCo‐LDH Coated the Different CNT_a_SBS_b_@F

The buckled porous carbon fabric samples (CNT_1_SBS_1_@F, CNT_2_SBS_1_@F, and CNT_3_SBS_1_@F) were fully dried, and they were subject to hydrothermal treatment to grow NiCo‐LDH nanostructures on the surface. The hydrothermal process was carried out at 120 °C for 12 h. The hydrothermal solution consisted of varying concentrations of cobalt (II) sulfate hexahydrate, nickel (II) sulfate hexahydrate, urea, and SDBS. Three different solutions were prepared: 20 mm CoSO₄·6H₂O, 20 mm NiSO₄·6H₂O, excess urea, and 0.1 wt.% SDBS; 20 mm CoSO₄·6H₂O, 40 mm NiSO₄·6H₂O, excess urea, and 0.1 wt.% SDBS; 40 mm CoSO₄·6H₂O, 20 mm NiSO₄·6H₂O, excess urea, and 0.1 wt.% SDBS, respectively. After the hydrothermal treatment, the resulting samples were labeled according to the NiCo‐LDH coating applied to the original porous carbon fabrics. The samples prepared with solution (20 mm CoSO₄·6H₂O, 40 mm NiSO₄·6H₂O) on the CNT_2_SBS_1_@F scaffold were named Ni_20_Co_40_ LDH@CNT_2_SBS_1_@F, where the total mass loading of NiCo‐LDH nanoflakes was ≈2 mg cm⁻^2^. The other samples were labeled as Ni_20_Co_20_ LDH@CNT_1_SBS_1_@F and Ni_40_Co_20_ LDH@CNT_3_SBS_1_@F, respectively.

### Fabrication of the All‐Solid‐State Symmetric Supercapacitor

The gel electrolyte was prepared by dissolving 2.5 g of acrylamide and 2.5 mg of BMAA in 10 mL of deionized water. This solution added 0.06 g of KPS and 5 µL of TEMED under constant stirring at room temperature. Once homogeneous, 10 mL of 6 m KOH solution was rapidly added to form a pre‐gel electrolyte solution. The prepared Ni_20_Co_40_‐LDH@CNT_2_SBS_1_@F electrodes were immersed in the gel electrolyte for 30 s to ensure uniform absorption. The samples were then allowed to solidify at 50 °C for 30 min. This process was repeated 2–3 times to ensure a uniform coating of the gel electrolyte on the fibers. After the final coating, the samples of two same‐coated gel Ni_20_Co_40_‐LDH@CNT_2_SBS_1_@F were assembled into symmetric supercapacitors and stored at room temperature for full solidification.

### Characterization

To comprehensively analyze the samples of NiCo@CNTSBS@F, CNTSBS@F, and fabrics, several characterization techniques were employed. Scanning electron microscopy (SEM) was performed using a Tescan MIRA field emission SEM to examine surface morphology, coupled with energy‐dispersive X‐ray spectroscopy (EDX) to map the elemental distribution. High‐resolution transmission electron microscopy (HRTEM) was conducted on a JEOL JEM‐2010 to observe nanostructures and lattice fringes. X‐ray photoelectron spectroscopy (XPS) was carried out with a Thermo Scientific Nexsa G2 system to analyze elemental composition and oxidation states. Nitrogen adsorption‐desorption isotherms were measured using a Micromeritics ASAP 2020 system. The prepared samples were degassed under vacuum at 120 °C for 12 h prior to analysis to remove adsorbed moisture and contaminants. BET surface area was determined from the nitrogen adsorption isotherms, while the pore size distribution was calculated using the BJH method based on the desorption branch of the isotherm. The XRD pattern was performed on a Bruker X‐ray diffractometer (D8 Advance) with Cu Kα radiation. The electrochemical performance of the fabricated supercapacitors (NiCo @CNTSBS@F, size 1 × 1 cm) and the all‐solid‐state symmetric supercapacitor were tested via an electrochemical workstation instrument (CHI 660E, shanghai chenhua instrument Co., Ltd.) The NiCo @CNTSBS@F samples as a working electrode were evaluated using a three‐electrode system in a 3 m KOH aqueous electrolyte. A graphite plate and a saturated calomel electrode served as the counter and reference electrodes, respectively. Cyclic voltammetry (CV), galvanostatic charge–discharge (GCD), and electrochemical impedance spectroscopy (EIS) were employed to characterize the capacitive behavior, rate performance, and impedance characteristics of the supercapacitor. The cycling stability of the supercapacitors was evaluated through continuous GCD cycling at 20 mA cm⁻^2^ for 5000 cycles and 20 000 cycles, in the gel and solution electrolytes. Specific capacitances were calculated from the GCD profiles using the following equation:

(1) $$C=\frac{I*\Delta t}{\Delta V}$$

where I is the discharge current (mA cm^−2^), Δt is the discharge time (s), and ΔV is the potential window(V).

The energy density (E) and power density (P) were calculated using the following equations:

(2) $$E=\frac{1}{2}\;*C*\frac{U^{2}}{3600}$$

(3) $$P=\frac{E*3600}{\Delta t}$$

where U and Δt are the voltage window (V) and the discharging duration (s), respectively.

## Conflict of Interest

The authors declare no conflict of interest.

## Supporting information



Supporting Information

## Data Availability

The data that support the findings of this study are available from the corresponding author upon reasonable request.
