# Supplementary material for: Hierarchically Porous Wearable Composites for High‐Performance Stretchable Supercapacitors
Source: Adv Sci (Weinh). 2025 Apr 25;12(25):2500835. doi: 10.1002/advs.202500835 (PMC12224955; doi:10.1002/advs.202500835)
Supplement: Supplementary file 1 — Supporting Information [file ADVS-12-2500835-s001.docx]

**Supporting Information**


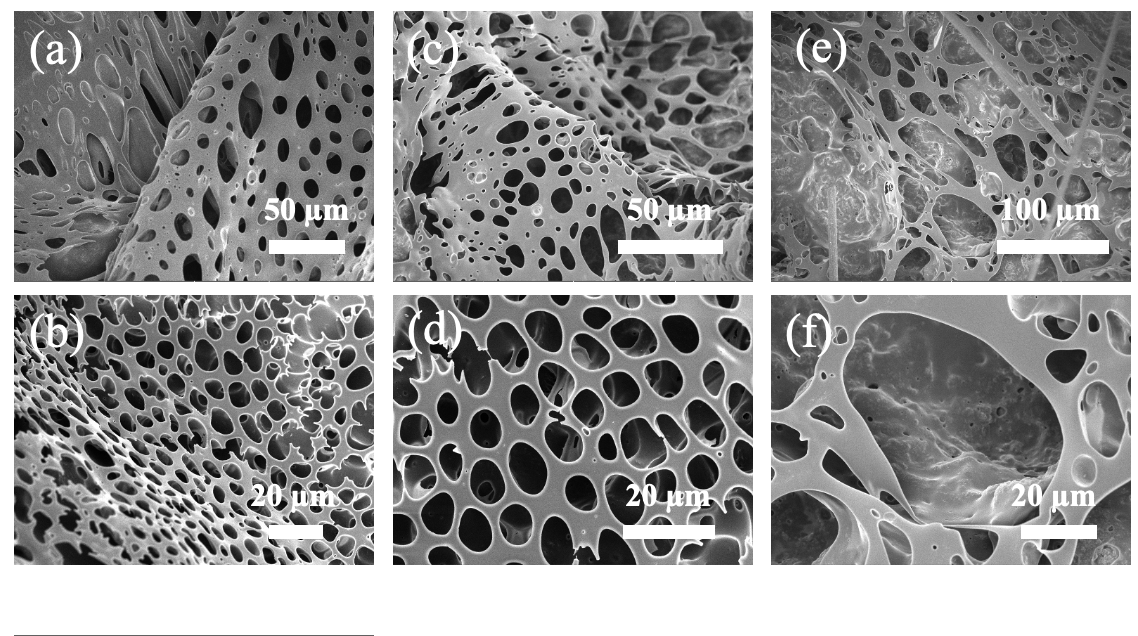


**Figure S1** SEM images of different SBS and CNT ratios solution coatings on carbon fabric substrates at varying magnifications. (a, b) the morphology of CNT_1_SBS_1_ @ F, (c, d) the morphology of CNT_2_SBS_1_ @ F, (e, f) the morphology of CNT_3_SBS_1_ @ F.


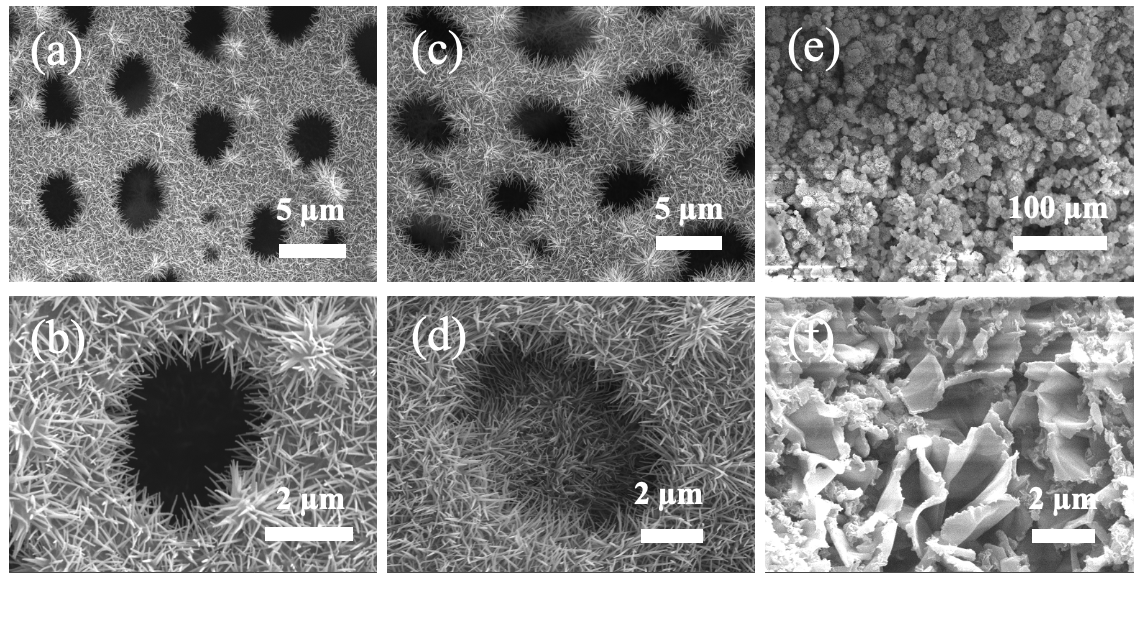


**Figure S2** Micromorphology characterization of the growth and distribution of NiCo-LDH under the CNT_2_SBS_1_@F hydrothermal different Ni₂SO₄ and Co₂SO₄ mixed solutions. (a, b) different magnified typical SEM image of Ni_20_Co_20_ LDH @ CNT_2_SBS_1_ @ F, (c, d) different magnified typical SEM image of Ni_20_Co_40_ LDH@CNT_2_SBS_1_@F, and (e, f) different magnified typical SEM image of Ni_40_Co_20_ LDH@CNT_2_SBS_1_@F.


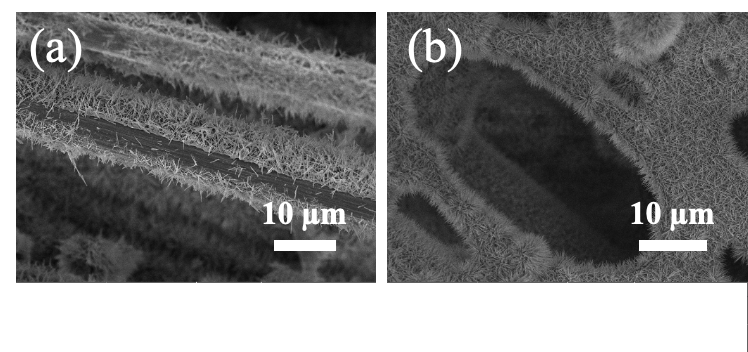


Figure S3 SEM images comparing the morphology of Ni_20_Co_40_ LDH grown on different substrates: (b) Ni_20_Co_40_ LDH@CNT_2_SBS_1_@F, and (a) Ni_20_Co_40_ LDH@F.


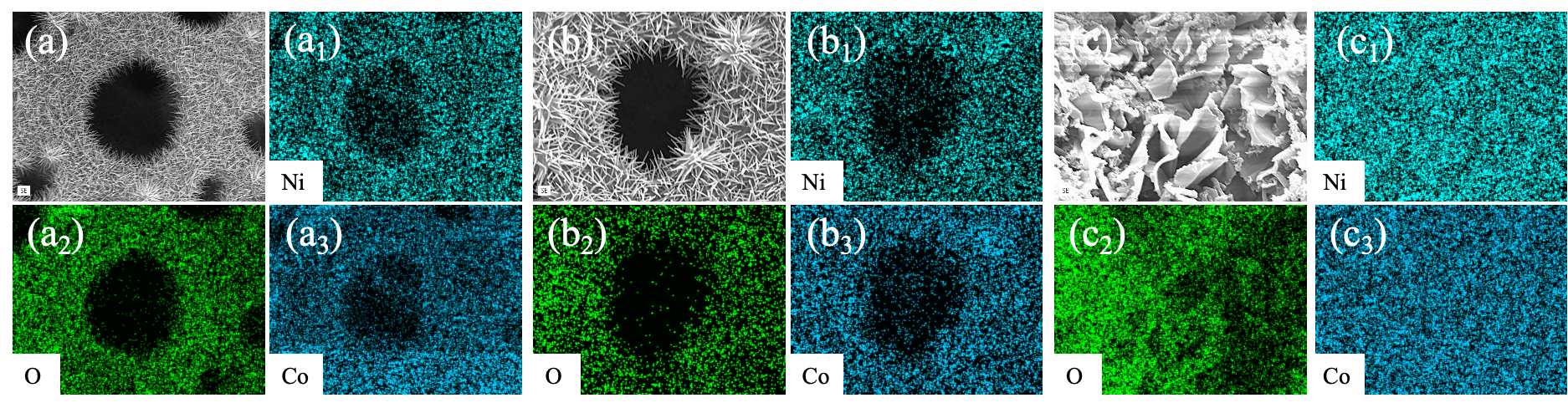


**Figure S4** EDX elemental mappings for the three samples: (a-a3) Ni_20_Co_20_ LDH@CNT_2_SBS_1_@F, (b-b3) Ni_20_Co_40_ LDH@CNT_2_SBS_1_@F, and (c-c3) Ni_40_Co_20_ LDH@CNT_2_SBS_1_@F.


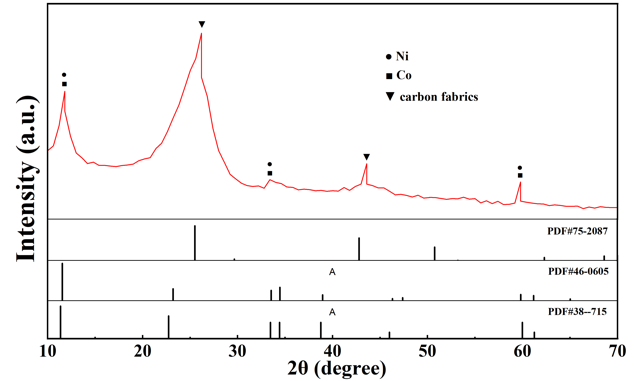


**Figure S5** X-ray diffraction (XRD) image of Ni_20_Co_40_ LDH@CNT_2_SBS_1_@F.


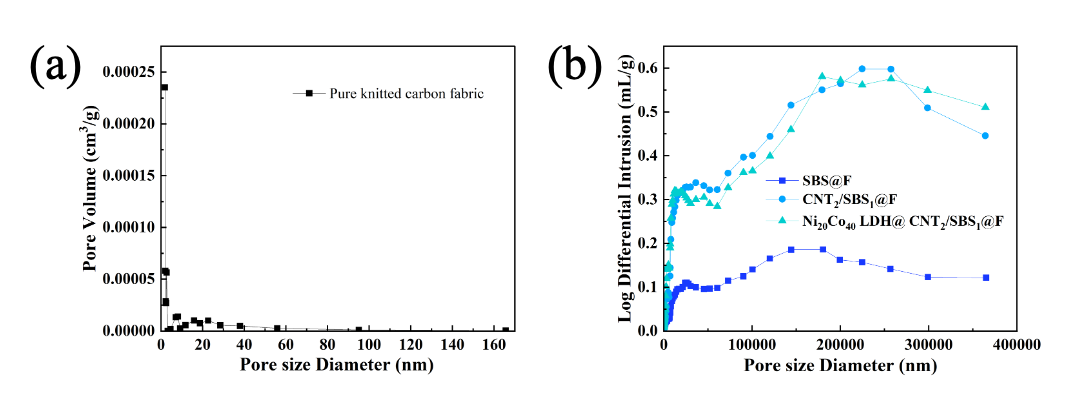


**Figure S6**. The BET analysis of pore size diameter with pure knitted carbon fabric (a), SBS@F, CNT_2_/SBS_1_@F and Ni_20_Co_40_ LDH@ CNT_2_/SBS_1_@F (b).


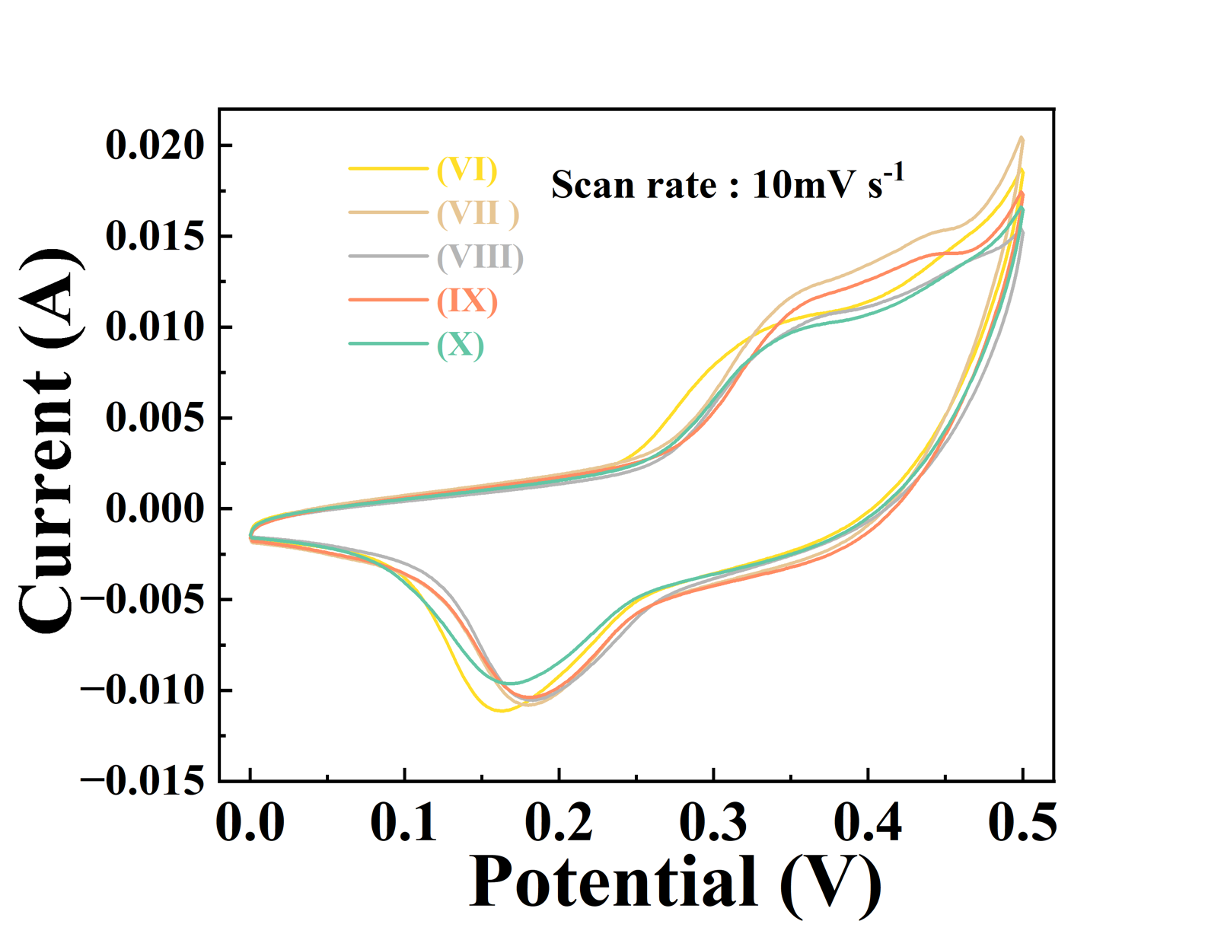


**Figure S7** Cyclic voltammetry (CV) curves of Samples VI-X at a scan rate of 10 mV s⁻¹.


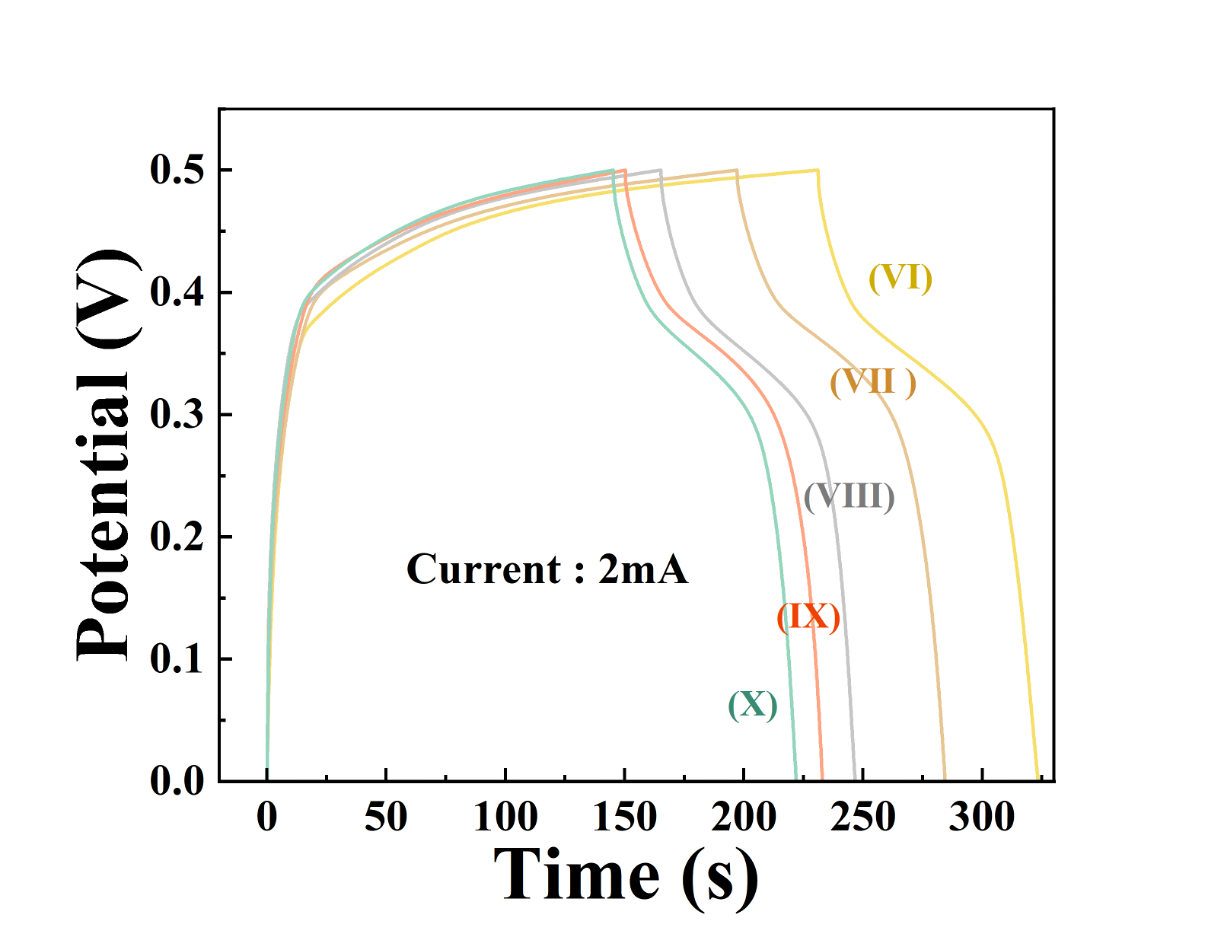


**Figure S8** Galvanostatic charge-discharge (GCD) curves of Samples VI-X at a 2 mA cm⁻² current density.


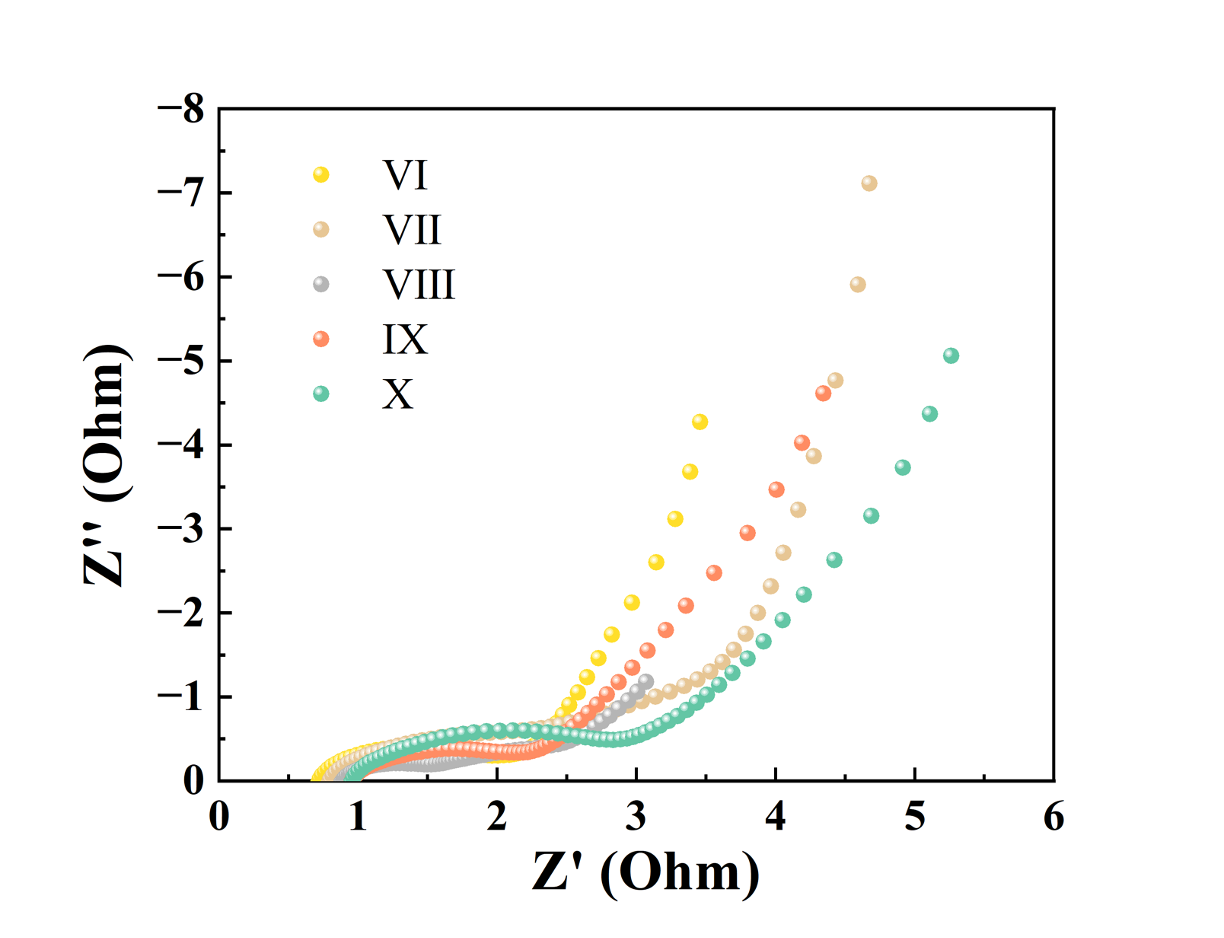


**Figure S9** Nyquist plots of Samples VI-X obtained from electrochemical impedance spectroscopy (EIS).


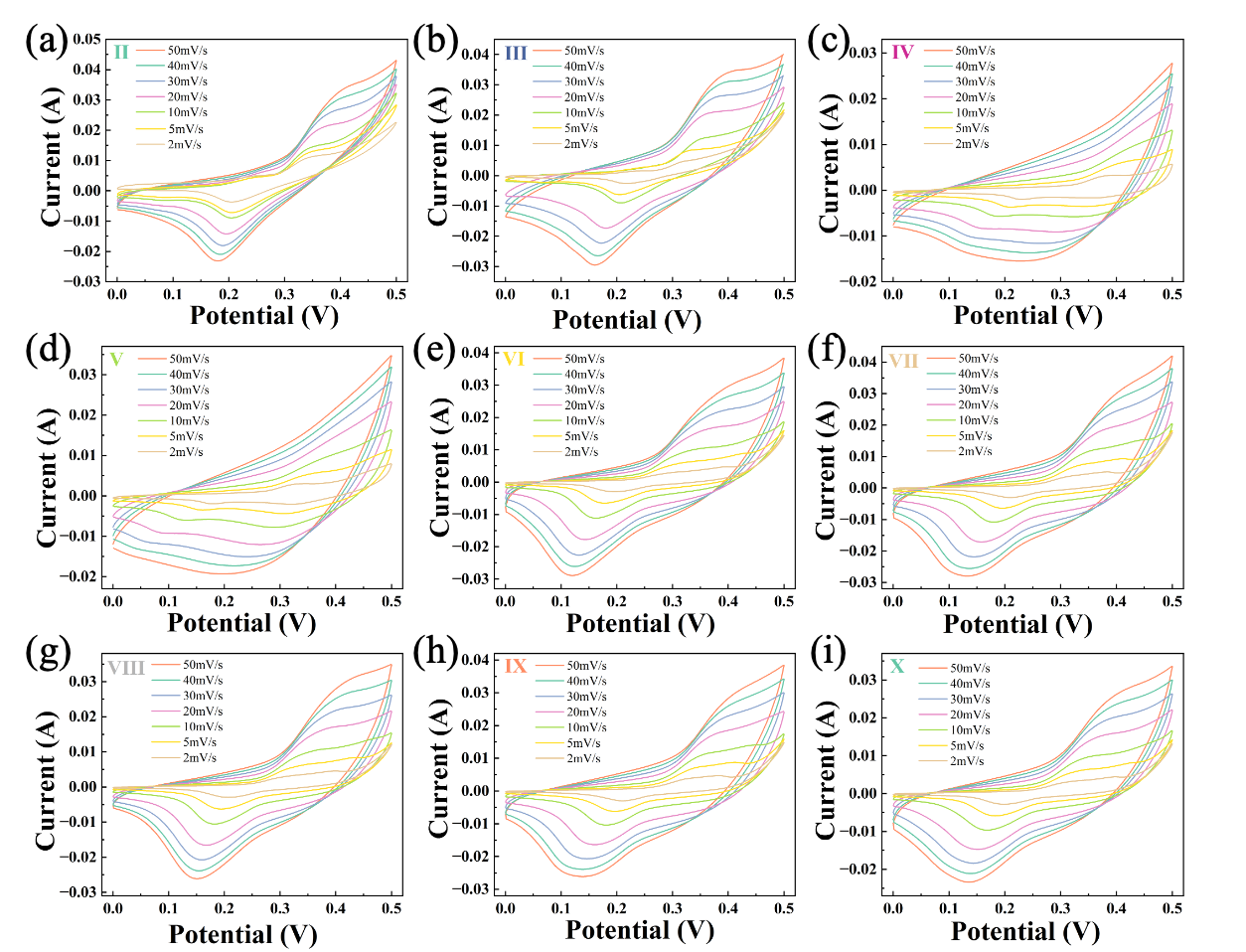


**Figure S10** Cyclic voltammetry (CV) curves of Samples II-X at various scan rates (2 mV/s to 50 mV/s).


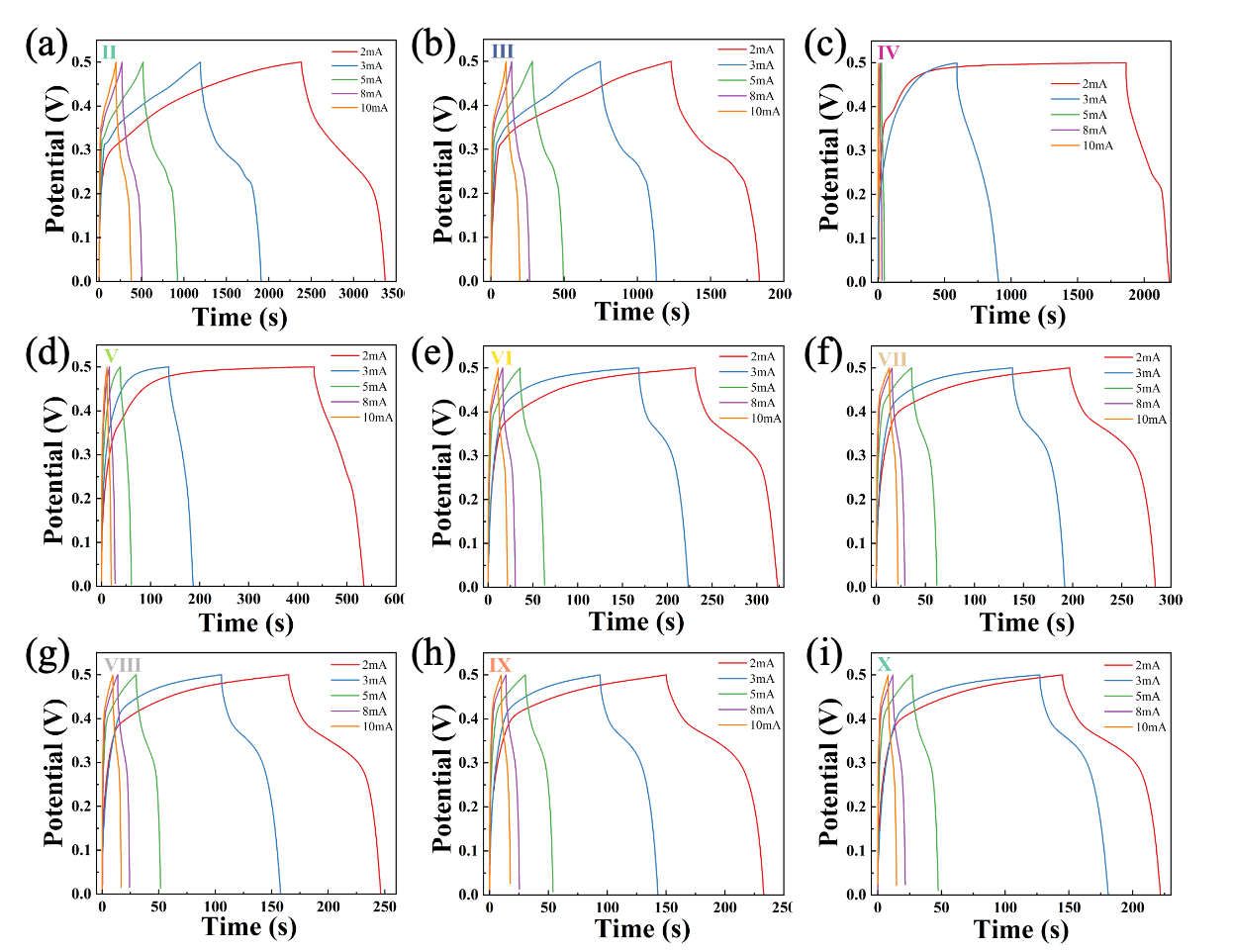


**Figure S11** Galvanostatic charge-discharge (GCD) curves of Samples II-X at different current densities (2, 3, 5, 8, and 10 mA cm⁻²).


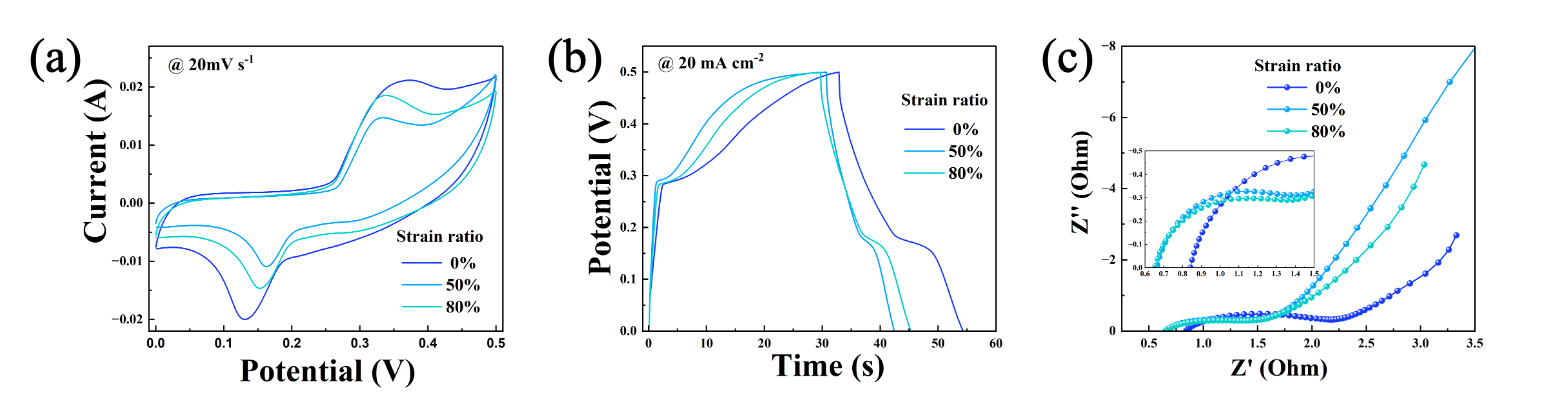


**Figure S12** Electrochemical performance of the Ni_20_Co_40_@CNT_2_SBS_1_@F in a three-electrode system under different tensile strains (0%, 50%, and 80%). (a) Cyclic voltammetry (CV) curves at a scan rate of 20 mV s⁻¹. (b) Galvanostatic charge-discharge (GCD) curves at a current density of 20 mA cm⁻². (c) Electrochemical impedance spectroscopy (EIS) Nyquist plots.

**
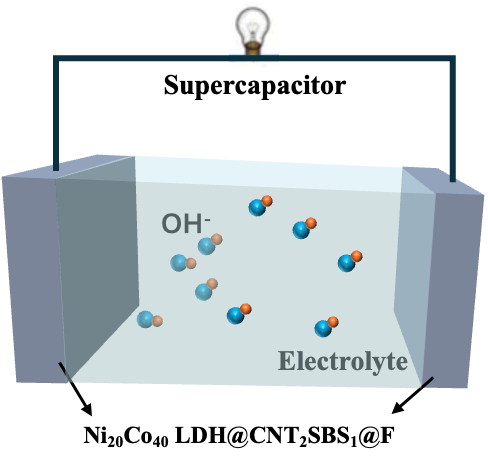
**

**Figure S13** Schematic diagram of the symmetric supercapacitor based on Ni_20_Co_40_ LDH@CNT_2_SBS_1_@F.


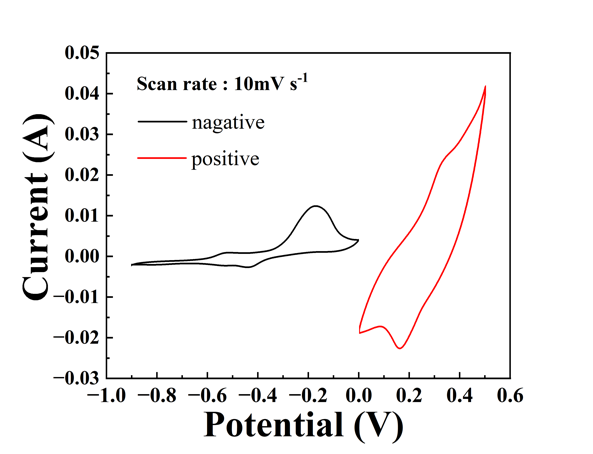


**Figure S14** CV curves of sample I as negative and positive


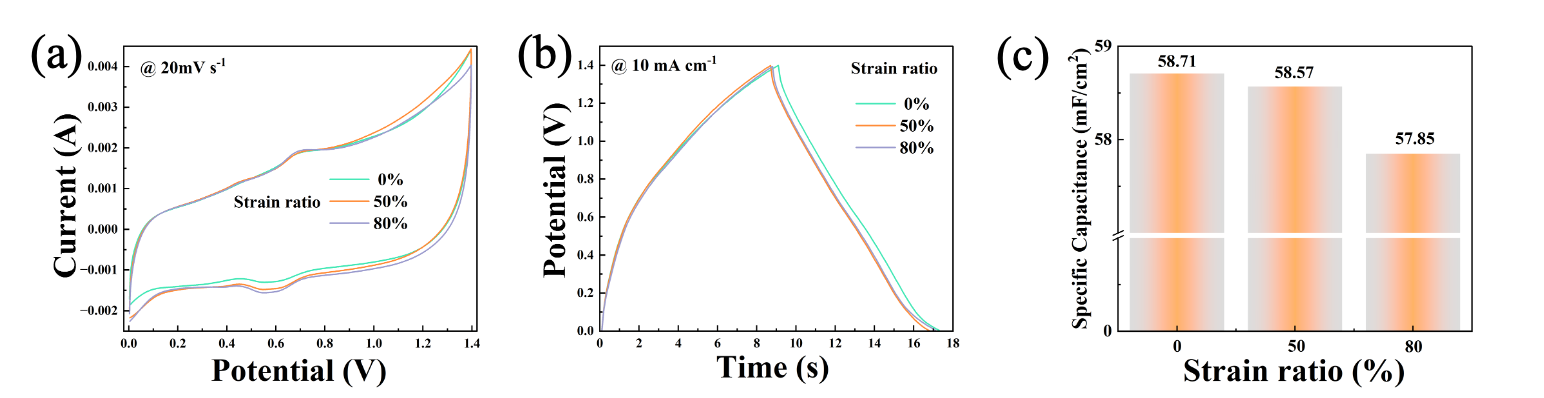


**Figure 15** Electrochemical performance of the stretchable supercapacitor under different tensile strains (0%, 50%, and 80%). (a) CV curves at a scan rate of 20 mV s⁻¹. (b) GCD curves at a current density of 10 mA cm⁻². (c) specific capacitance.


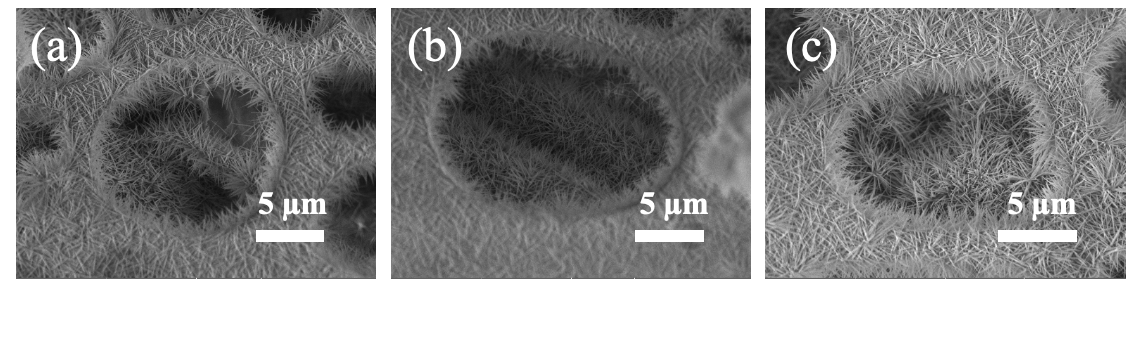


**Figure S16** SEM images of Ni_20_Co_40_@CNT_2_SBS_1_@F electrodes after being stretched to different strain ratio: (a) 0% strain, (b) 50% strain, and (c) 80% strain.


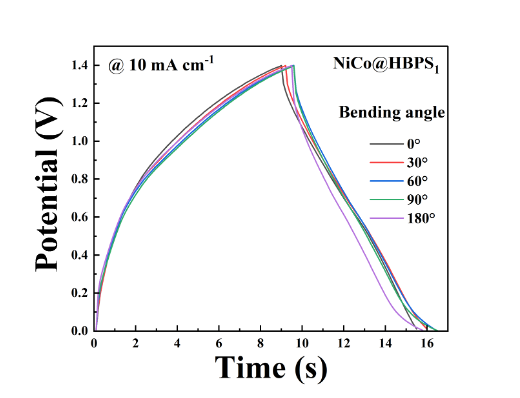


**Figure S17** GCD curves of the supercapacitor at a current density of 10 mA cm⁻² under different bending angles (0°, 30°, 60°, 90°, and 180°)


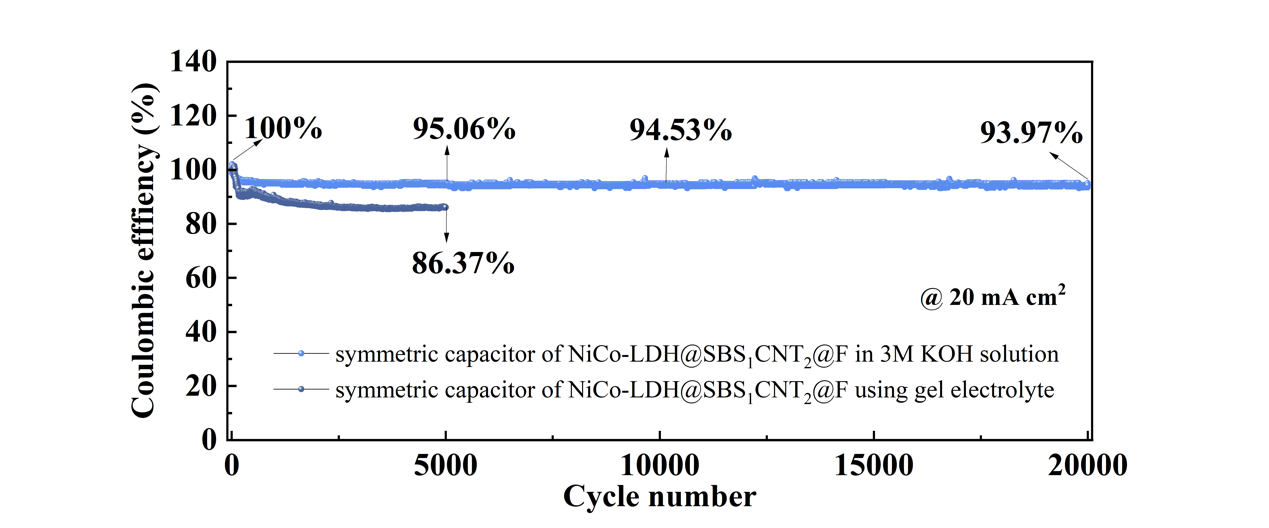


**Figure S18** Long-term cycling performance of asymmetric capacitor of Ni_20_Co_40_ LDH@CNT_2_SBS_1_@F in different condision.


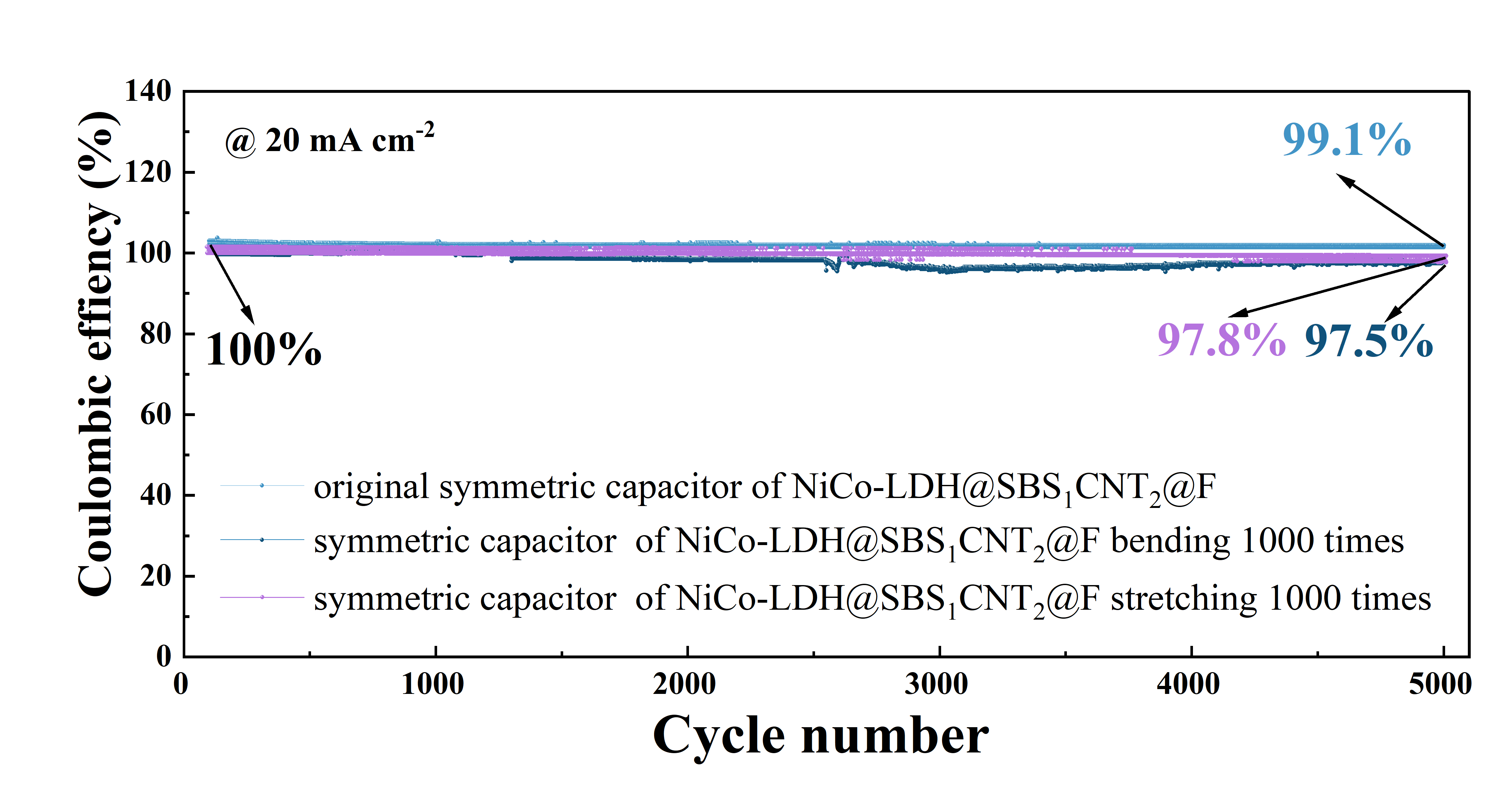


**Figure S19** Coulombic efficiency of the symmetric supercapacitors (original device and 1000 times repeated bending and 1000 times repeated stretching) measured at 20 mA cm⁻² over 5000 charge-discharge cycles.

**Table S1** Comparison of NiCo materials used as active materials for supercapacitors.

| **LDH materials** | **Capacitance** | **Stability (Cycle)** | **Energy Density (E)** | **Power Density (P)** |  | **Ref.** |
| --- | --- | --- | --- | --- | --- | --- |
| 3D NiCo-LDH microflowers derived from ZIF-67 | 1765 F g⁻¹ at 1 A g | 84.3% retention after 6000 cycles | 53.31 Wh kg⁻¹ | 749.7 W kg⁻¹ |  | [37] |
| Al-doped NiCoP derived from NiCo-LDH precursors | 1059 F g⁻¹ at 1 A g⁻¹ | 99% retention after 10000 cycles | 51.7 Wh kg⁻¹ | 500 W kg⁻¹ |  | [38] |
| Graphene Quantum Dots (GQDs) pinned on NiCo-LDH hollow micro-tunnels | 1628 F g⁻¹ at 1 A g⁻¹ | 86.2% retention after 8000 cycles | 46 Wh kg⁻¹ | 7440 W kg⁻¹ |  | [39] |
| NiCo-LDH microspheres derived from Ni-MOF | 1272 C g⁻¹ at 2 A g⁻¹ | 103.9% retention after 5000 cycles | 36.1 Wh kg⁻¹ | 649 W kg⁻¹ |  | [40] |
| NiCo-LDH nanosheet array/Ag nanowire | 2920.6 F g⁻¹ at 5 A g⁻¹ | 89.8% retention after 2000 cycles | 42.9 Wh kg⁻¹ | 800 W kg⁻¹ |  | [41] |
| NiCo-LDH nanowires@nanosheets core-shell | 9.67 F cm⁻² at 5 mA cm⁻² | 75% retention after 2000 cycles | 298.6 µWh cm⁻² | 0.80 mW cm⁻² |  | [42] |
| Yarn coated with Ni-Co-S | 127.2 mF cm^−2^ at 0.1 mA/cm | 90% retention after 3000 cycles | 48.7 µWh cm⁻² | 0.553mW cm⁻² |  | [43] |
| Ni(OH)_2_ nanosheet wrapped NiCo_2_O_4_ on CNTF | 291.9 mF cm^−2^ at 1 mA/cm^2^ | 95% retention after 5000 cycles | 103.8 µWh cm⁻² | 0.8 mW cm⁻^2^ |  | [44] |
| 3D hierarchical NiCo_2_O_4_@NiCo-LDH | 6092 mF cm^-2^ | 83.3% retention after 2000 cycles | 49 Wh kg^-1^ | 750 W kg^-1^ |  | [45] |
| **NiCo-LDH nanowires** | **4948 mF cm⁻² at 2 mA cm⁻²** | **93.97% retention after 20000 cycles** | **801.6µWh cm⁻² /400.5 Wh kg⁻¹** | **3.5mW cm⁻² /1749.5 W kg⁻¹** |  | **This work** |

**Table S2** Comparison of different electrode materials for stretchable supercapacitors.

| **Active Material** | **Reversible elongation (%)** | **Capacitance** | **Energy Density (E)** | **Power Density (P)** | **Ref.** |
| --- | --- | --- | --- | --- | --- |
| CNT | 30% | 7.3F g^-1^ at 0.25A g^-1^ | 1.4 Wh kg^-1^ | 0.9kW kg^-1^ | [46] |
| V_2_O_5_/PEDOT | 50% | 135mF cm^-2^ at 0.5mA cm^-2^ | 1.3mWh cm^-3^ | 188 mW cm^-3^ | [47] |
| LIG/PDMS | 50% | 650mF cm^-2^ at 35mA cm^-2^ | / | / | [48] |
| Graphene/MoS_2_ composite | 60% | 70mF cm-2 | 1.728mWh cm^-2^ | 0.062W cm^-3^ | [49] |
| PANI/PVA | 50% | 11.3 mF cm^-2^ at 5mV s^-1^ | / | / | [50] |
| Graphene sheet | 60% | 5.8µF cm-2 at 0.1V s^-1^ | / | / | [51] |
| 3D-PEDOT:PSS/ graphene | 50% | 19.3 mF cm^−2^ at 20 mV s^−1^ | 52.6 Wh kg^-1^ | 19.1 kWkg | [52] |
| SWCNT/SBS nanofibre | 40% | 15.2 F cm^−3^ at 0.021 A cm^−3^ | 2 mWh cm^−3^ | 158 mW cm^−3^ | [53] |
| PANI/rGO | 40% | 112 F g^−1^ at 0.08 A g^−1^ | 8.80 mWh cm^−3^ | 30.77 mW cm^−3^ | [54] |
| MnO_2_/CNT core–shell | 40% | 61.25 mF cm^−2^ at 10 mV s^−1^ | 5.5 µWh cm^−3^ | / | [55] |
| **NiCo-LDH nanowires** | 80% | **4948 mF cm⁻² at 2 mA cm⁻²** | **801.6µWh cm⁻² /400.5 Wh kg⁻¹** | **3.5mW cm⁻² /1749.5 W kg⁻¹** | **This work** |
